# Natural‐Inspired Engineered Microalgae With Selenium Enrichment Enabled Potent Anti‐Tumor Immunity

**DOI:** 10.1002/advs.77297

**Published:** 2026-08-27

**Authors:** Yuqi Yang, Xiaowei Cai, Xiaoyan Zhong, Tian Qiu, Qinwei Lu, Jiahao Qin, Fei Gong, Yecheng Huang, Chenyu Jiang, Youdong Chen, Yuan Cheng, Liang Cheng

**Affiliations:** ^1^ Institute of Functional Nano & Soft Materials (FUNSOM) Jiangsu Key Laboratory of Advanced Healthcare Materials & Devices Biomedical‐BasicResearch‐Center (BBRC) of Jiangsu Province Soochow University Suzhou China; ^2^ Suzhou Industrial Park Monash Research Institute of Science and Technology Monash University Suzhou China; ^3^ Department of Materials Science and Engineering Faculty of Engineering Monash University Clayton Australia; ^4^ Department of Urology The Second Affiliated Hospital of Soochow University Suzhou China; ^5^ Department of Toxicology School of Public Health Suzhou Medical College of Soochow University Suzhou China

**Keywords:** bladder cancer, immune checkpoint blockade, immunotherapy, microalgae, selenium

## Abstract

Immunotherapy has shed light on clinical oncology for several decades. However, it still suffers from heterogeneous therapeutic outcomes and potential immune‐related adverse events (irAEs). Inspired by a natural phenomenon that microalgae can absorb and accumulate selenium (Se), a well‐known healthcare element, we engineered a Se‐enriched cyanobacterium *Synechococcus Sp* (Se‐*Ssp*) for advanced immunotherapy. Simply by culturing in the presence of Na_2_SeO_3_, Se nanoparticles could be bio‐fabricated within *Synechococcus Sp*, underscoring a facile preparation process with promising translational potential. With satisfactory biocompatibility and internalization efficiency, Se‐*Ssp* boosted dendritic cell maturation and antigen presentation via both the Toll‐like receptor and the cyclic GMP‐AMP synthase‐stimulator of interferon genes (cGAS‐STING) pathways, likely attributed to the Se‐enhanced influx of calcium. In vivo studies demonstrated that Se‐*Ssp* elicited potent innate immunity and maintained long‐term adaptive immunity, effectively inhibiting tumor growth with sustained protection against recurrence. In addition, Se‐*Ssp* was validated to sensitize the reaction of both primary and distant tumors to immune checkpoint and indoleamine 2,3‐dioxygenase inhibitors; it also restrained the progression of orthotopic bladder carcinoma via intravesical instillation as well. Briefly, this study developed an anti‐cancer immune application of engineered Se‐*Ssp*, offering a promising alternative with significant therapeutic potential that might benefit patients with cancer.

## 1 Introduction

Cancer immunotherapy has played an increasingly important role in clinical oncology over the past decades [1]. The arsenal of approved immunotherapies continues to expand rapidly, with prominent examples including adoptive cell therapies and immune checkpoint blockade (ICB) [2, 3]. However, this progress remained limited by suboptimal response rates, unpredictable interpatient efficacy, and the risk of immune‐related adverse events (irAEs), ranging from manageable autoimmune reactions to severe, life‐threatening complications [4, 5]. The wide variation in tumor response rates to immunotherapy is related to tumor heterogeneity and abnormal metabolism, the presence of an immunosuppressive “cold” tumor microenvironment (TME), and the potential occurrence of resistance and immune escape [6, 7, 8, 9]. Improving antigen presentation and T cell infiltration with enhanced functions to convert “cold” tumors into “hot” is one of the efficient solutions that can be established by various approaches [9, 10, 11, 12, 13]. Among these, employing microorganisms such as bacteria, viruses, and microalgae for microbial cancer immunotherapy displayed unique therapeutic functions with sustainable modulation, reshaping the immune system markedly in the long term [14, 15, 16, 17]. For example, therapy involving the gut microbiota and its specific metabolites reportedly improved anti‐cancer immunity to control distant tumors [18, 19]. However, live microbes enable potent immunomodulation but carry risks such as cytokine storms and uncontrolled colonization, while attenuated forms lose a part of their therapeutic performance; therefore, resolving this dilemma requires further decoration of microorganisms [14].

Selenium (Se) is an essential trace element widely used in dietary supplements and health products [20, 21]. Studies have shown that adequate Se intake enhances immune functions, particularly in regulating immune cell activity, as exemplified by the ability of Se supplementation to fuel T cell proliferation and tumor cytotoxicity [22]. Notably, here is an interesting environmental phenomenon — algae can absorb Se as either selenite (SeIV, Se_2_O_3_) or selenate (SeVI, Se_2_O_4_) in water, which has already been used in wastewater treatment and biorefinery [23, 24]. Some cyanobacteria, including *Spirulina platensis*, *Phormidium luridum* var. *olivacea*, and *Anacystis nidulans* syn. *Synechococcus leopoliensis* can generate elemental Se granules under high Se stress [25]. What are the possible roles of Se‐enriched microalgae in cancer immunotherapy? These naturally engineered microalgae are a harmonious blend of Se and microorganisms, which might feature potential therapeutic performance in combating cancer. Moreover, cyanobacteria are relatively safe, as they proliferate more slowly than typical fast‐growing bacteria to reduce the risk of uncontrolled infection and systemic toxicity; in addition, these kinds of microorganisms possess a modified form of lipopolysaccharide (LPS) that is structurally distinct from the conventional LPSs found in Gram‐negative bacteria, further alleviating the toxicity and being expected to be used in clinical applications [26, 27, 28]. However, even though the theory is valid, this particular area remains largely unexplored.

Inspired by this natural engineering, we filled a critical void in the development of Se‐enriched *Synechococcus Sp* (Se‐*Ssp*) through the easy and simple biosynthesis of Se nanoparticles (Se NPs) in *Synechococcus Sp* (*Ssp*, a generally considered safe strain) for immunomodulatory applications. Se‐*Ssp* was effectively internalized by cells, resulting in satisfactory maturation promotion of dendritic cells (DCs) by activating not only the Toll‐like receptor (TLR) pathway but also the cyclic GMP‐AMP synthase‐stimulator of interferon genes (cGAS‐STING) pathway, which was possibly attributed to Se‐enhanced calcium (Ca) influx. Based on these findings, intratumoral injection of Se‐*Ssp* primed robust immunity in CT26 tumor‐bearing mice, thereby realizing potent tumor inhibition; the cured mice were rechallenged with cancer cells, while there was no obvious recurrence. Mechanistic studies based on transcriptomic analysis further uncovered that Se‐*Ssp* triggered innate immunity and regulated adaptive immunity via multiple pathways, transforming the “cold” TME into “hot” to facilitate tumor elimination. Furthermore, such Se‐enriched microalgae sensitized tumors to the clinically used immune checkpoint inhibitor (ICI) anti‐cytotoxic T‐lymphocyte antigen 4 (αCTLA‐4) antibody and even the clinically failed indoleamine 2,3‐dioxygenase (IDO) inhibitor NLG919, as evidenced by both suppressed primary and distant tumors. Notably, Se‐*Ssp* was applied to perfusion therapy to treat orthotopic bladder cancer, where it even displayed superior immunostimulatory effects compared with the clinical standard Bacillus Calmette‐Guérin (BCG) vaccine at the tested dosage. Briefly, this study highlighted the potential of Se‐enriched microalgae as potent anti‐tumor immunotherapeutic agents; the observed sensitization to both clinically relevant ICIs and previously ineffective IDO inhibitors underscored the potential of these microalgae agents to augment the efficacy of existing cancer therapies, representing a promising alternative for advanced oncotherapy (Scheme 1).

**SCHEME 1:**
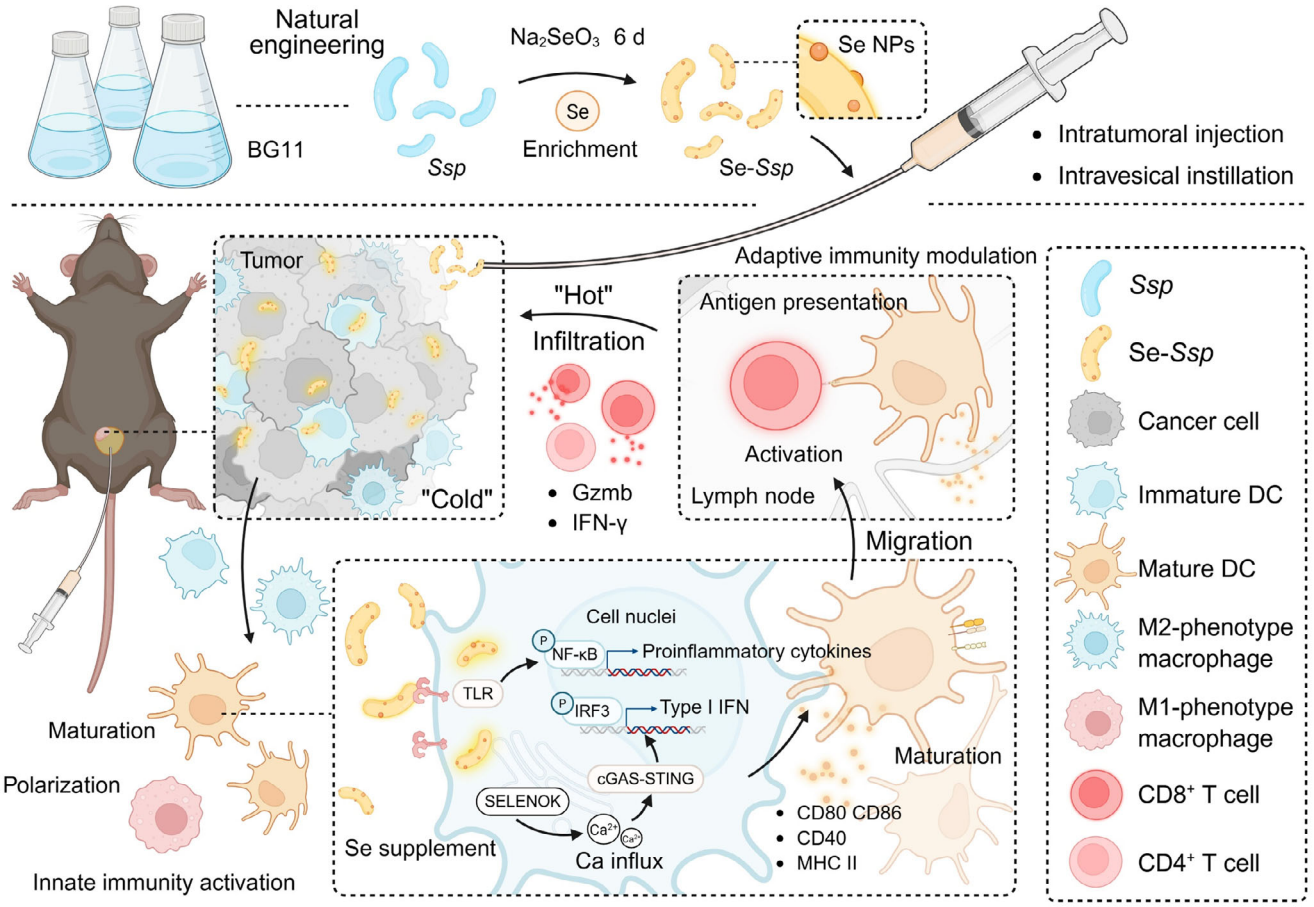
Se‐*Ssp* was engineered as a potent immunomodulator for activating innate immunity via the TLR and cGAS‐STING pathways, therefore converting “cold” tumors into “hot” tumors to achieve high efficacy and long‐term therapeutic outcomes.

## 2 Results and Discussion

### 2.1 Preparation and Characterization of Se‐*Ssp*

*Ssp* were cultured under Se stress for 8 days (Figure 1a). According to the photographs and the ultraviolet‐visible absorption spectra, the color of *Ssp* changed from normal blue‐green to orange, with the absorption peaks of three main photosynthetic pigments in cyanobacteria disappearing, including carotenoids (at around 420 nm), phycocyanin (at around 620 nm), and chlorophyll a (at around 670 nm) (Figure 1b) [25]. As no significant changes of spectra were observed after 6 days, the products after 6‐day engineering were selected for subsequent investigation. This induction protocol was scalable as well (Figure S1a). Coomassie Brilliant Blue (CBB) was used to visualize the protein profiles of *Ssp* before and after Se exposure; the bands revealed the degradation of most proteins, such as phycocyanin, except for those located at 50–70 kDa on the gel (Figure 1c). Based on the protein profiling analysis results, these bands corresponded to some membrane proteins and the large subunit of ribulose‐1,5‐bisphosphate carboxylase/oxygenase (RuBisCO), which enables microalgae to fix carbon dioxide during photosynthesis for the subsequent synthesis of proteins, lipids, nucleic acids, etc (Figure S1b) [29]. Moreover, after exposure to high‐level Se, the Se concentration of the harvested product, namely, Se‐*Ssp*, increased by 7.9 µg per 10^7^ cells, indicating Se bioaccumulation within this kind of microalgae; scanning electron microscopy (SEM), transmission electron microscopy (TEM) images, and dynamic light scattering (DLS) revealed that although the size and morphology did not obviously change, some nanoscale particles were found to be located on Se‐*Ssp* (Figure 1d‐g, Figure S1c). This surface change might also contributed to the variation in the zeta potential (Figure 1h). After purification, these orange nanoparticles from Se‐*Ssp* were collected and determined to be Se(0) nanoparticles (NPs) via element mapping, energy dispersive spectrometry (EDS), and X‐ray photoelectron spectroscopy (XPS) analysis, which was consistent with the previous studies showing that *Ssp* could generate Se NPs when exposed to a high‐Se environment (Figure 1i, j). The surface of these Se NPs showed C, O, N, and S signals, and exhibited a negative zeta potential (Figure S1d‐h). The bicinchoninic acid (BCA) assay confirmed the presence of protein (Figure S1i). Together, these findings indicated that a protein corona possibly formed on the surface of Se NPs [25]. Considering the reduction reaction of the transition from Se(IV) in Na_2_SeO_3_ to Se(0), two reducing agents, including ascorbic acid (AA) and glutathione (GSH), at physiological concentrations in microalgae, were applied to simulate the synthesis process to some extent [30]. The solution turned orange with a significant Tyndall effect in the mixed solution of Na_2_SeO_3_ and GSH, while there were no visible changes in response to AA addition. It is known that inter‐thylakoid spaces contain abundant GSH (Figure 1k) [31]. Interestingly, the nanoscale Se product reduced by GSH (labeled as G‐Se NPs) was very similar in size (approximately 50 nm) to the Se NPs purified from Se‐*Ssp* (Figure 1l). In addition, no obvious color and spectra changes could be observed when *Ssp* were cultured in the dark, indicating that Se accumulation and assimilation were a light‐requiring active process (Figure S1j) [25, 32]. These results implied that the generation of Se NPs might be related to the GSH‐based reduction reaction within *Ssp* under light illumination.

**FIGURE 1:**
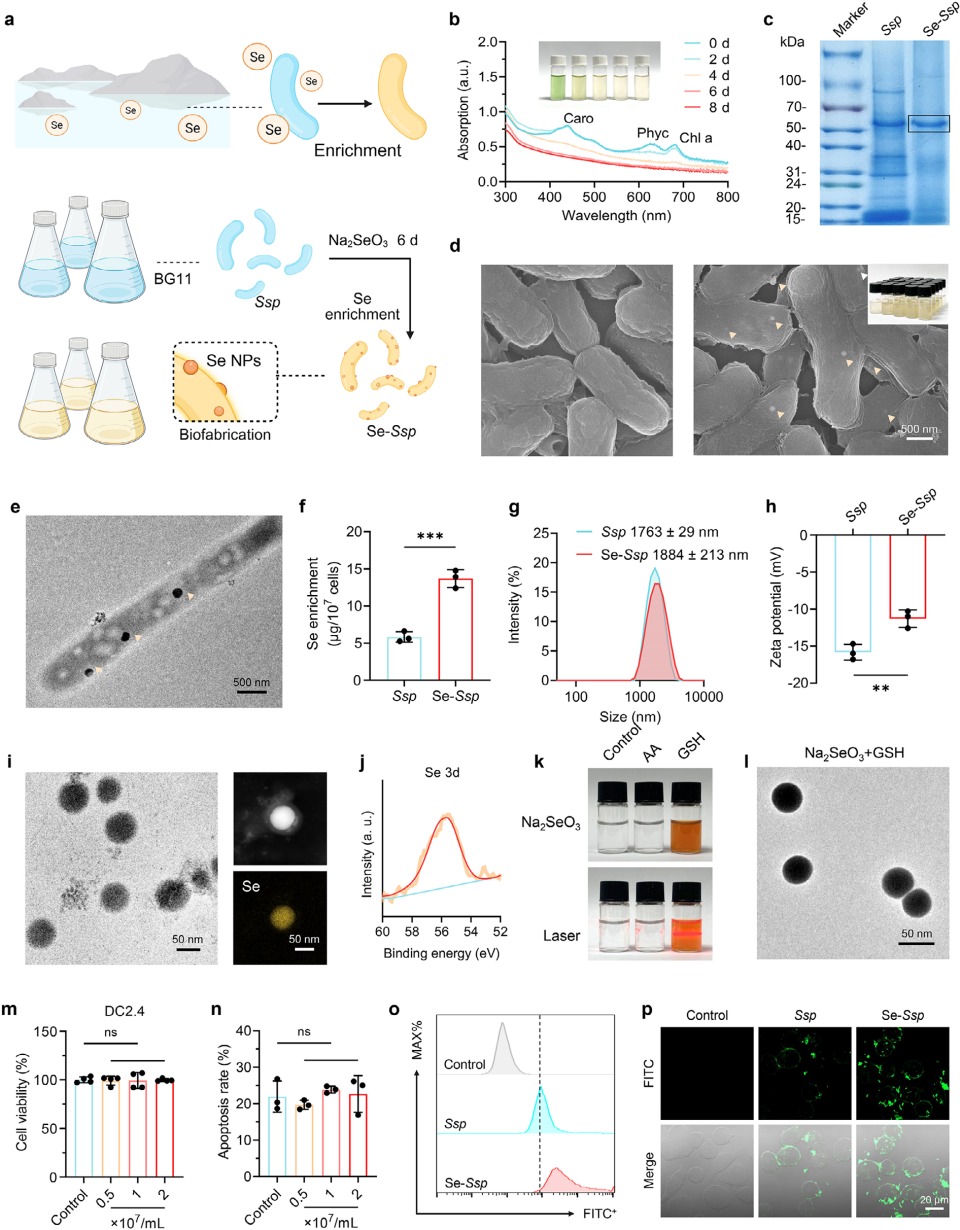
Characterization and biocompatibility of Se‐*Ssp*. (a) Inspiration and schematic illustration of the Se‐enrichment of Se‐*Ssp*. (b) The optical absorption of *Ssp* cultured in BG11 containing Na_2_SeO_3_ on days 0, 2, 4, 6, and 8. (c) CBB staining of the supernatants of *Ssp* and Se‐*Ssp*. (d) SEM images of *Ssp* and Se‐*Ssp*. (e) TEM image of Se‐*Ssp* with Se NPs. (f) Se concentrations in *Ssp* and Se‐*Ssp*. (g) The hydrodynamic sizes of *Ssp* and Se‐*Ssp*. (h) Zeta potentials of *Ssp* and Se‐*Ssp*. (i) Purified Se NPs and elemental mapping. (j) XPS spectra of the purified Se NPs. (k) Image of the Se NP simulation, with the Tyndall effect by laser illumination. (l) TEM image of Se NPs synthesized from Na_2_SeO_3_ and GSH. (m) Relative cell viability of DC2.4 cells incubated with Se‐*Ssp* for 12 h (n = 4). (n) Annexin V‐FITC^+^ apoptosis rate of DC2.4 cells incubated with Se‐*Ssp* for 12 h (n = 3). (o) Representative histogram of the FITC signal in DC2.4 cells incubated with *Ssp* or Se‐*Ssp* for 12 h. (p) Representative fluorescence images of DC2.4 cells incubated with *Ssp* and Se‐*Ssp* for 6 h. Data are presented as the mean ± SD. Statistical significance was calculated via unpaired two‐tailed Student's t‐test and expressed as ns *p* > 0.05, *p < 0.05, **p < 0.01, and ***p < 0.001.

### 2.2 Biocompatibility of Se‐*Ssp*

Considering the immune‐modulating effects of both microalgae and Se, Se‐*Ssp* was hypothesized to serve as a promising immunotherapeutic candidate for oncotherapy. We then studied the biocompatibility of Se‐*Ssp* on DC2.4 cells, a murine DC line, via the standard methylthiazolyldiphenyl‐tetrazolium bromide (MTT) assay. Se‐*Ssp* exhibited minimal cytotoxicity even at a dose of 2×10^7^ /mL (Figure 1m). Furthermore, compared with that in the untreated group, only a slight increase in the percentage of apoptotic DC2.4 cells was detected (Figure 1n). Similar favorable biocompatibility was also found in the murine macrophage line RAW264.7 and human umbilical vein endothelial cells (HUVECs); for RAW264.7 cells, a slight proliferation occurred when the cells were subjected to a low dosage of Se‐Ssp (Figure S1k). On the other hand, Se‐*Ssp* inhibited a degree of cell proliferation when tested across several cancer cell lines, such as the murine colorectal cancer cell line CT26, the murine prostate cancer cell line RM‐1, the murine mammary cancer cell line 4T1, and the murine bladder cancer cell line MB‐49 (Figure S1l); this vulnerability to Se might be related to the fact that tumor cells typically exhibit elevated basal levels of reactive oxygen species (ROS) and rely heavily on antioxidant systems, while Se exposure further disrupted this balance and led to the reduced viability [33, 34]. In addition to good compatibility with biological systems of healthy cells, efficient cellular internalization is a prerequisite for achieving potent efficacy. Thus, *Ssp* and Se‐*Ssp* were fluorescently labeled with fluorescein isothiocyanate (FITC), followed by being co‐cultured with DC2.4. According to the flow cytometry and confocal laser scanning microscopy (CLSM) images with semi‐quantification of FITC, Se‐*Ssp* were phagocytosed by DC2.4 more promptly, possibly because of its higher zeta potential and rougher surface texture than those of *Ssp* (Figure 1o,p, Figure S1m). Briefly, these observations and data demonstrated that Se‐*Ssp* possessed biosafety within a certain range, along with an enhanced cellular uptake efficiency relative to that of *Ssp*.

### 2.3 In Vitro Immunostimulatory Effects of Se‐*Ssp*

Since Se‐*Ssp* contains immunogenic compounds from microorganisms, along with Se NPs showing potential relative effects, it is expected to trigger a bunch of immune‐related outcomes. As a priority, antigen‐presenting cells (APCs) capture, process, and present antigens to facilitate subsequent adaptive immune responses; among these, DCs are widely recognized as the pioneers of professional APCs that play a pivotal role in anti‐tumor immunity. Thus, mouse bone marrow‐derived DCs (BMDCs) were first employed to evaluate the effects of Se‐*Ssp*. While *Ssp* moderately improved the maturation of BMDCs, Se‐*Ssp*‐treated BMDCs presented satisfactory expression levels of the co‐stimulatory molecules CD80 and CD86 (∼ 80.5%), even surpassing those of BMDCs subjected to 500 ng/mL LPS (Figure 2b,c). Similarly, Se‐*Ssp* promoted the CD40 expressionefficiently on BMDCs (Figure S2b). Moreover, exposure to Se‐*Ssp* induced a significant upregulation of major histocompatibility complex class II (MHC II) on the surface of BMDCs, thereby increasing their antigen‐presenting capacity (Figure 2d, Figure S2a). The secretion of pro‐inflammatory cytokines is a kind of reliable indicator of DC activation; as anticipated, co‐culture with Se‐*Ssp* indeed increased the release of tumor necrosis factor α (TNF‐α), interleukin 6 (IL‐6), and interleukin 1β (IL‐1β) from BMDCs into the media (Figure 2e). Similar trends can be observed in DCs when they are incubated with the microalgal debris rather than intact *Ssp* or Se‐*Ssp*, despite the efficacy being relatively moderate (Figure S2c‐e). Macrophages, another important type of APCs, were subjected to Se‐*Ssp* to further demonstrate remarkable immunopotentiation. As evidenced by the higher ratio of the M1‐phenotype to the M2‐phenotype in RAW 264.7 cells, Se‐*Ssp* could modulate inflammatory polarization to a greater extent than *Ssp* did (Figure S2f‐h). Overall, these results suggested that the Se‐containing microalgae Se‐*Ssp* could exert potent immunostimulatory effects, at least on two important kinds of APCs, highlighting their great potential in combating cancer.

**FIGURE 2:**
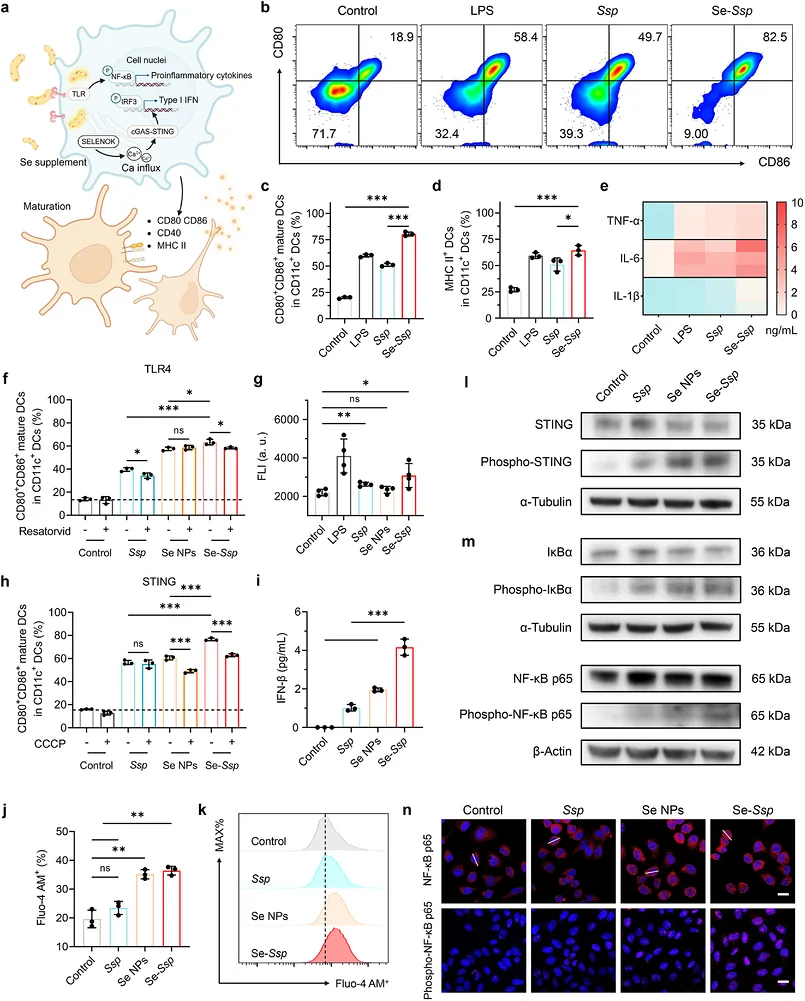
In vitro immune stimulatory effects of Se‐*Ssp*. (a) Schematic illustration of immune activation induced by Se‐*Ssp*. (b) Representative flow dot plots of DC maturation stimulated with LPS, *Ssp*, and Se‐*Ssp*. (c) The quantification of DC maturation stimulated with LPS, *Ssp*, and Se‐*Ssp* (n = 3). (d) The quantification of antigen presentation of DC stimulated with LPS, *Ssp*, and Se‐*Ssp* (n = 3). (e) ELISA results of TNF‐α, IL‐6, and IL‐1β in the culture media of DCs (n = 3). (f) The quantification of DC maturation stimulated with *Ssp*, Se NPs, and Se‐*Ssp*, with or without pre‐treatment with Resatorvid as a TLR4 inhibitor (n = 3). (g) The fluorescence intensity (FLI) of HEK‐Dual mTLR4 (NF/IL‐8) cells stimulated with *Ssp*, Se NPs, and Se‐*Ssp* by using QUANTI‐Luc as the detection reagent (n = 3). (h) The quantification of DC maturation stimulated with *Ssp*, Se NPs, and Se‐*Ssp*, with or without pre‐treatment with CCCP as a cGAS‐STING inhibitor (n = 3). (i) ELISA results of IFN‐β in the culture media of DCs (n = 3). (j) The quantification of Fluo‐4 AM^+^ DC2.4 cells incubated with *Ssp*, Se NPs, and Se‐*Ssp* (n = 3). (k) Representative histogram of the Fluo‐4 AM^+^ signal in DC2.4 cells incubated with *Ssp*, Se NPs, and Se‐*Ssp*. (l, m) Western blotting analysis of (l) STING and phospho‐STING, (m) IκBα, phospho‐IκBα, NF‐κB p65, and phospho‐NF‐κB p65 proteins in DC2.4 cells incubated with *Ssp*, Se NPs, and Se‐*Ssp*. (n) Representative immunofluorescence images of the NF‐κB p65, and phospho‐NF‐κB p65 expression in DC2.4 cells. Scale bar: 20 µm. Data are presented as the mean ± SD. Statistical significance was calculated via unpaired two‐tailed Student's t‐test and expressed as ns *p* > 0.05, *p < 0.05, **p < 0.01, and ***p < 0.001.

In light of these intriguing results, the possible mechanisms underlying the effects of Se‐*Ssp* were explored. We initially investigated whether the intracellular change in ROS level induced by microalgae or Se, as a mild level of ROS is beneficial for APC maturation, whereas excessive ROS will impair APC function and induce oxidative damage [35, 36]. However, no obvious difference could be found in the control, *Ssp*, Se NPs, and Se‐*Ssp* groups at this low dosage — this suggests that the immunostimulatory effect of Se‐*Ssp* was not strongly associated with ROS changes (Figure S3b). Given that cyanobacteria share structural and compositional similarities with Gram‐negative bacteria in triggering innate immune responses, we first explored whether Se‐*Ssp* could stimulate the TLR pathways, especially TLR4, which can recognize LPS or LPS‐like secondary metabolites [15, 37]. Taking TLR4 as a model, the selective TLR4 signaling inhibitor Resatorvid was then used to assess the TLR4‐dependent capacity of Se‐*Ssp* [38]. The decreased maturation levels indicated that Se‐*Ssp* inherited the TLR4‐activating function of *Ssp*, whereas the purified Se NPs showed no significant effect (Figure 2f). By applying murine TLR4 dual reporter HEK293 cells transfected with the mouse TLR4 (mTLR4) MD‐2/CD14 genes, the engagement of TLR4 signaling in response to *Ssp* and Se‐*Ssp* was further validated (Figure 2g) [39]. Hence, Se‐*Ssp* could promote TLR4 activation at least.

In addition to this, the cGAS‐STING pathway is another important inducer of DC maturation. Interestingly, treatment with one of the cGAS‐STING inhibitors, carbonyl cyanide 3‐chlorophenylhydrazone (CCCP), significantly suppressed the proportion of mature DCs induced by Se NPs and Se‐*Ssp* (Figure 2h) [40]. For the detailed process of this pathway, activated STING undergoes conformational change and translocates from the endoplasmic reticulum to the Golgi, where it recruits and activates TANK‐binding kinase 1 (TBK1). Activated TBK1 phosphorylates interferon regulatory factor (IRF3), leading to its dimerization and nuclear translocation, ultimately inducing type I interferon (IFN) expression [41, 42, 43]. As expected, increased levels of IFN‐β, a cytokine closely regulated by STING, were detected in the culture media of the DCs in these two Se‐stimulating groups (Figure 2i) [14]. The protein levels of phospho‐STING, phospho‐TBK1/NAK, and phospho‐IRF3 improved, especially when the samples were subjected to Se‐*Ssp* (Figure 2l, Figure S3a). These three notable findings indicated that Se potentially played the role of a trigger or promoter of the cGAS‐STING axis. Based on this, several Se sources were used to activate DC. The results showed that Na_2_SeO_3_ inhibited DC maturation; the G‐Se NPs synthesized with Na_2_SeO_3_ and GSH had only marginal effects on promoting the expression of CD80 and CD86, while the Se NPs and Se‐*Ssp* still featured remarkable stimulatory potencies (Figure S3c). The differential immunopotentiation among the G‐Se NPs, Se NPs, and Se‐*Ssp* might be attributed to the higher zeta potential and existing surface biomacromolecules of Se NPs and Se‐*Ssp*, strongly supporting the unique advantages of Se‐enriched microalgae obtained via the nature‐inspired inducible synthesis approach for immunotherapeutic applications (Figure S3d). Mechanistically, we hypothesized that this phenomenon was probably linked to the supplementation of specific selenoproteins in immune cells, such as selenoprotein K (SELENOK), a protein localized in the endoplasmic reticulum membrane that is involved in Ca flux and is critical for innate immune responses; emerging evidence suggests that such selenoproteins might stabilize STING while promoting its oligomerization, thereby influencing TBK1 activation and downstream IFN‐β production [44, 45]. As expected, compared with *Ssp*, treatment with Se NPs and Se‐*Ssp* resulted in elevated levels of SELENOK, accompanied by an amplified signal of Fluo‐4 AM, a well‐established Ca indicator (Figure S3e, Figure 2j,k). The L‐type Ca channel antagonist Nifedipine was also found to partially reduce the percentage of mature DC in these two groups with Se, but it did not significantly affect the control or *Ssp* groups. This indicated that Se from Se‐*Ssp* could promote Ca influx and further make a contribution to STING activation (Figure S3f). Collectively, while retaining the capacity to facilitate TLR4 pathways from *Ssp*, Se‐enriched Se‐*Ssp* was also able to supply Se to immune cells by up‐regulating selenoprotein (at least SELENOK), thus simultaneously priming the cGAS‐STING pathway (Figure 2a). Notably, the immunostimulatory effects of Se‐*Ssp* were much better than those of LPS, Mn^2+^, and even the combination of LPS and Mn^2+^, which were well‐characterized agonists of the TLR4 and cGAS‐STING pathways, respectively (Figure S3g) [14, 39]. These outcomes ultimately boosted the phosphorylation and subsequent degradation of IκBα, releasing the nuclear factor κB (NF‐κB) transcription factor that then translocated into the nucleus and underwent phosphorylation to initiate a series of robust inflammatory responses (Figure 2m,n, Figure s3h,i) [46, 47].

### 2.4 Potent Anti‐Tumor Immunity Triggered by Se‐*Ssp*

A systematic characterization of the in vivo immunomodulation by Se‐*Ssp* was further performed. CT26‐bearing mice were randomly divided into four groups, three of which were intratumorally injected with *Ssp* (10^7^ cells), Se NPs (10 µg), or Se‐*Ssp* (10^7^ cells) three times; these mice were sacrificed on day 3 or day 7 after the last administration for the collection of inguinal lymph nodes (recognized as tumor‐draining lymph nodes, TDLNs) and tumors to analyze the immune cell populations (Figure 3a). The injections triggered innate immunity in the early stage. While the DC maturation level was only modestly elevated in the *Ssp* group, more CD80^+^CD86^+^ DCs were detected in the TDLN samples from the Se NPs and Se‐*Ssp* groups (Figure 3b,d). We then subdivided the DCs into CD11c^+^MHCII^high^ migratory DCs (MigDCs) and CD11c^+^MHCII^low^ resident DCs (ResDCs) [48]. The MigDCs migrated from inflamed tumor sites, which were significantly higher‐degree mature (reaching more than 30%) in the Se NPs and Se‐*Ssp* groups (Figure 3e); these activated MigDCs migrating to TDLNs also stimulated ResDCs to initiate subsequent immunity cooperatively (Figure S4a). However, the ability of the less mature MigDCs to modulate the ResDCs seemed a little weak in the *Ssp* group. Interestingly, even on day 7 after the last administration, unlike the population that dropped closely to the blank level in the *Ssp* group, the proportions of DC maturation in the Se NPs and Se‐*Ssp* groups were still significantly greater than those in the control group, indicating that more sustained immunostimulatory effects were mediated by Se (Figure 3i).

**FIGURE 3:**
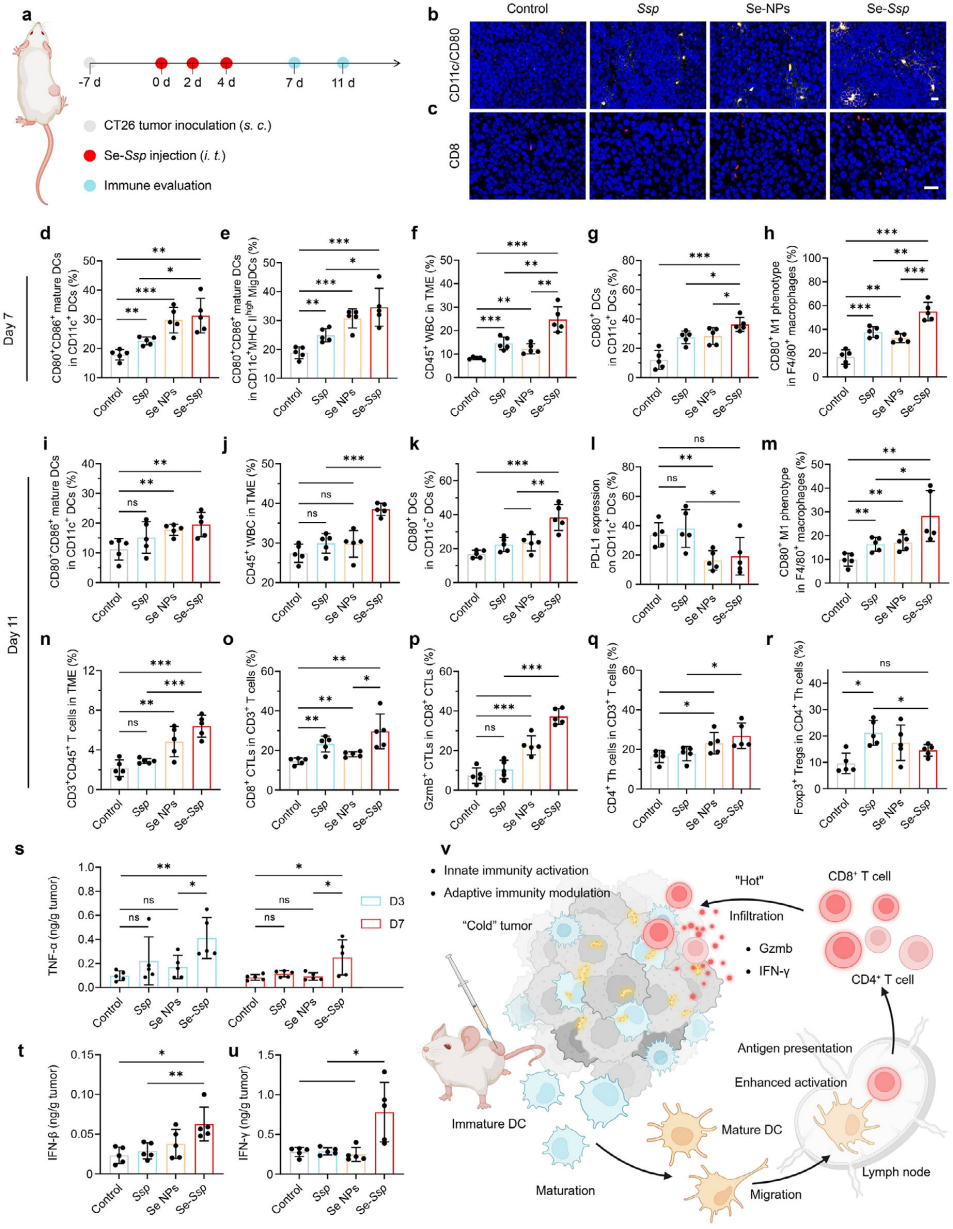
In vivo immune evaluation based on intratumoral injection of Se‐*Ssp* in the CT26 subcutaneous tumor model. (a) Schematic illustration and timeline of Se‐*Ssp* intratumoral injection and immune evaluation of the CT26 colon tumor in Balb/c mice. (b‐t), Groups: control (n = 5), *Ssp* (n = 5), Se NPs (n = 5), and Se‐*Ssp* (n = 5). (b, c) Representative immunofluorescence images of (b) CD11c/CD80 and (c) CD8 of tumor sections. (d‐h) the quantification of (d) DC maturation, (e) MigDC maturation in TDLNs, the percentages of (f) lymphocytes, (g) DC maturation, and (h) M1‐phenotype macrophages in tumors on day 3 after the last injection. (i‐r) the quantification of (i) DC maturation in TDLNs, the percentages of (j) lymphocytes, (k) DC maturation, (l) PD‐L1 expression on DCs, (m) M1‐phenotype macrophages, (n) T cells, (o) CTLs, (p) GzmB generation in CTLs, (q) Th cells, and (r) Tregs in tumors on day 7 after the last injection. (s‐u) the ELISA results for (s) TNF‐α in tumors on day 3 and day 7 after the last injection, (t) IFN‐β in tumors on day 3 after the last injection, and (u) IFN‐γ in tumors on day 7 after the last injection. (v) Schematic illustration of the process of in vivo immunomodulation induced by Se‐*Ssp*. Scale bar: 20 µm. Data are presented as the mean ± SD. Statistical significance was calculated via unpaired two‐tailed Student's t‐test and expressed as ns *p* > 0.05, *p < 0.05, **p < 0.01, and ***p < 0.001.

At the tumor site, injecting these three agents enhanced lymphocytic infiltration, which increased by ∼ 6.42%, ∼ 4.08%, and ∼ 16.6%, respectively (Figure 3f). From this point of view, *Ssp* resulted in better lymphocyte recruitment than Se NPs did; this might be due to its microscale size, which was beneficial for longer retention at tumor sites. When the tumors were treated with Se‐*Ssp*, the percentage of CD80‐expressing DCs was approximately 3‐fold greater than that of the control; as demonstrated previously, this credit went to the concurrent initiation of the TLR and cGAS‐STING pathways (Figure 3g). Such Se‐*Ssp* injection also promoted increased infiltration of immune cells and activation of DCs, as evidenced by the stable, significant results on day 11 (Figure 3j,k). Interestingly, the expression of programmed cell death ligand 1 (PD‐L1), an immune checkpoint on DCs from the Se NPs and Se‐*Ssp* groups, was reduced at the same time compared with that in the *Ssp* group, indicating that Se biofortification in microalgae might relieve immune tolerance to some extent (Figure 3l). Moreover, consistent with the trend of lymphocytic infiltration, the proportion of macrophages within the treated tumor also increased at the initial stage (Figure 3h). Although the effect of Se NPs was less impressive, there still were more macrophages polarized to the pro‐inflammatory M1‐phenotypes than to the anti‐inflammatory M2‐phenotype in the groups subjected to *Ssp* and Se‐*Ssp*, particularly Se‐*Ssp* (Figure S4b). Surprisingly, Se‐*Ssp* injection sustained the robust infiltration and M1‐polarization of macrophages even on day 11 (Figure 3m), although the M2‐phenotype percentages slightly increased (Figure S4f).

T lymphocytes play a crucial role in cancer immunotherapy. For these cells, only a slight increase was found after 3 days of treatment with *Ssp* or Se‐*Ssp*, indicating that adaptive immunity might not have yet been activated at this stage (Figure S4c‐e). On day 11, the percentages of T cells increased to 4.8% and even 6.4% in both the Se NPs and Se‐*Ssp* groups, respectively, while the percentage in the *Ssp* group did not obviously differ from that in the control group (Figure 3c,n, Figure S4g). Although *Ssp* and Se‐*Ssp* improved the differentiation toward CD8^+^ cytotoxic T lymphocytes (CTLs) (Figure 3o), Se NPs and Se‐*Ssp* promoted the generation of Granzyme B (GzmB, a serine protease that can induce apoptosis in cancer cells) in CTLs, demonstrating that the bioaccumulation of Se in microalgae strongly enhanced anti‐cancer adaptive immunity (Figure 3c,p) [49, 50]. These Se‐augmented effects were also reflected in the increase in CD4^+^ Th cells in the Se NPs and Se‐*Ssp* groups (Figure 3q, Figure S4h). Notably, Se‐*Ssp* induced only a slight increase in Treg cells, which led to less immune tolerance than *Ssp* did from this perspective (Figure 3r).

Enzyme‐linked immunosorbent assay (ELISA) was then conducted to test the supernatants from the tumor tissues at the same time. Pro‐inflammatory cytokines such as TNF‐α and IL‐6 were both significantly up‐regulated after the administration of Se‐*Ssp*, and this effect seemed more pronounced in the initial phase (Figure 3s, Figure S4i). The concentration of IFN‐β, a product of cGAS‐STING pathway activation, was also increased by Se‐*Ssp*, with the measurement on day 7 (Figure 3t). Moreover, the secretion of IFN‐γ, one of the core immune regulators that induces cancer cell apoptosis and necroptosis, was enhanced significantly only by Se‐*Ssp* (Figure 3u) [39]. In contrast, the expression of interleukin 10 (IL‐10), an anti‐inflammatory cytokine, was down‐regulated to some extent on day 11 (Figure S4j). These data conclusively revealed that Se‐*Ssp* primed potent innate immunity and modulated subsequent adaptive immunity to turn “cold” TME into “hot”, indicating that Se‐*Ssp* has great oncolytic potential for eliminating tumors (Figure 3v).

### 2.5 Satisfactory Therapeutic Outcomes of Se‐*Ssp*

Given its remarkable capacity to stimulate immune responses, the anti‐tumor efficacy of Se‐*Ssp* was further investigated in CT26‐bearing Balb/c mice. The blood from Balb/c mice was first used to test the hemolysis, with negative results indicating good biosafety in terms of blood compatibility even at a high dosage (Figure S5a). Next, CT26‐bearing Balb/c mice were randomly divided into four groups, including control, intratumorally injected with *Ssp* (10^7^ cells), Se NPs (10 µg), and Se‐*Ssp* (10^7^ cells) three times (Figure 4a). During the observation period, intratumoral injection of *Ssp* inhibited tumor growth only slightly, according to the curves. Se NPs indeed delayed tumor progression in two out of five mice; unfortunately, tumor recurrence still occurred, ultimately leading to mortality. Notably, Se‐*Ssp* inhibited tumor growth satisfactorily with less recurrence than Se NPs did; in fact, these three treatments resulted in some tumor shrinkage and even complete eradication, resulting in a 7/10 survival rate within two months (Figure 4b‐d, Figure S5b). This remarkable potency could be attributed to the combination of the intrinsic properties of *Ssp* and Se NPs via the ingenious process of natural engineering. Consistent with the overall trend in therapeutic performance, hematoxylin and eosin (H&E) staining of tumor sections revealed modest changes in the *Ssp* and Se NPs groups but noticeable tissue necrosis in the Se‐*Ssp* group (Figure 4e). TdT‐mediated dUTP nick‐end labeling (TUNEL) immunohistochemistry (IHC) of tumors subjected to the Se‐*Ssp* revealed an extensive brown area, indicating a greater proportion of apoptotic cancer cells (Figure 4f). On the contrary, Se‐*Ssp*‐treated tumors showed a weaker positive signal for Ki‐67, a proliferation‐related marker (Figure 4g). There was no significant change in mouse body weight, which showed the safety of the treatments to some extent (Figure S5c). Together with the impressive results of the immune evaluation, these observations suggested that the immunomodulation mediated by Se‐*Ssp* not only induced oncolytic effects but also disrupted the proliferation of tumor cells.

**FIGURE 4:**
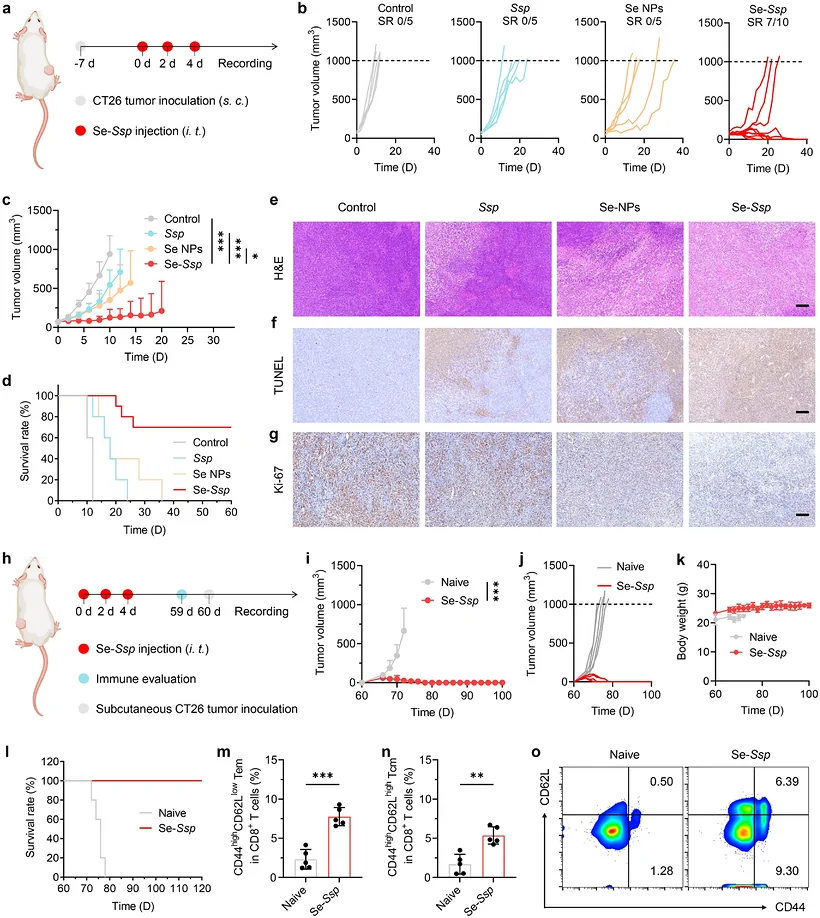
In vivo therapeutic outcomes based on the intratumoral injection of Se‐*Ssp* in the CT26 subcutaneous tumor model. (a) Schematic illustration and timeline of Se‐*Ssp* intratumoral injection into the CT26 colon tumors on Balb/c mice. (b‐g) Groups: control (n = 5), *Ssp* (n = 5), Se NPs (n = 5), and Se‐*Ssp* (n = 10). (b) The tumor volumes of individual mice. (c) The average tumor volume curves. (d) The overall survival rates. (e) Representative H&E staining of tumor sections. (f, g) Representative immunohistochemical images of (f) TUNEL and (g) Ki‐67 of tumor sections. (h) Schematic illustration and timeline of rechallenge in the CT26 colon tumors on Balb/c mice. (i‐o) Groups: naive (n = 5) and Se‐*Ssp* (n = 5). (i) The average tumor volume curves. (j) The tumor volumes of individual mice. (k) The average body weights. (l) The overall survival rates. (m, n) the quantification of (m) Tem and (n) Tcm in CTLs. (o) Representative flow dot plots of Tem and Tcm in CTLs. Scale bar: 100 µm. Data are presented as the mean ± SD. Statistical significance was calculated via unpaired two‐tailed Student's t‐test and expressed as ns *p* > 0.05, *p < 0.05, **p < 0.01, and ***p < 0.001.

These promising outcomes prompted us to assess the immune memory effects elicited by Se‐*Ssp*. On day 59 after the first treatment, the peripheral blood samples were collected from healthy mice (namely naïve) and surviving mice in the Se‐*Ssp* group to analyze the proportion of memory T cells (Figure 4h). As anticipated, the percentages of effector memory T cells (Tem) and central memory T cells (Tcm) within the CD8^+^ CTL population of the treated mice were approximately 3.4 and 3.2‐fold higher, respectively, than those in naïve mice (Figure 4m‐o). Similarly, the levels of Tem and Tcm in the CD4^+^ Th cells were also improved (Figure S5d‐f). Thanks to this remarkable immune memory, there was no obvious tumor regrowth when these mice were rechallenged with CT26 cells on day 60 (Figure 4i‐k). In addition, their survival time was prolonged to more than 120 days, without apparent variations in body weight (Figure 4l). In contrast, naïve mice experienced rapid tumor growth, demonstrating the benefits of Se‐*Ssp*‐induced long‐term immunity once again, which provided a robust theoretical foundation for its application in highly efficient oncologic immunotherapy.

### 2.6 In Vivo Immune Mechanisms of Se‐*Ssp*

Based on its powerful immunostimulatory effects and impressive therapeutic outcomes, transcriptome sequencing analysis of tumors from the control and Se‐*Ssp* groups was performed on day 5 after the last treatment (Figure 5a). A volcano plot revealed 1033 up‐regulated and 50 down‐regulated differential gene expressions (DEGs) between the Se‐*Ssp*‐treated group and the control group (Figure 5b); these DEGs were associated mainly with expression clusters related to differentiation markers, chemokines, and cytokines, along with some proteins involved in apoptosis and necrosis (Figure 5c). Gene Ontology (GO) enrichment analysis of these DEGs was then conducted, with 29 strongly correlated pathway entries shown in the bar graph, which could be summarized into four main parts: immune initiation (including both innate and adaptive immune responses), inflammation and cytokine signaling, cell migration and chemotaxis, and cell death via apoptosis and necrosis (Figure 5d). In addition, according to Kyoto Encyclopedia of Genes and Genomes (KEGG) pathway enrichment, crucial immune pathways, such as the Toll‐like receptor signaling pathway, the NF‐kappa B signaling pathway, antigen processing and presentation, and the T cell receptor signaling pathway, were primed; the Ca signaling pathway related to the cGAS‐STING enhancement was also enhanced by Se‐*Ssp* (Figure 5e). In line with the in vitro hypothesis and verification, Se‐*Ssp* was phagocytosed by APCs first, followed by boosting the NF‐κB cascade explicitly, and then antigen presentation to T cells to modulate adaptive responses; the network of inflammatory cytokines and chemokines was strengthened as well, not only acting directly on tumor cells, but also mobilizing the immune system to generate synergistic killing. Interestingly, other processes were also involved in this immune process. These include natural killer cell‐mediated cytotoxicity that could further eliminate tumor cells directly and provide an important defense against malignancies [51]. The enhanced B cell receptor signaling pathway also contributed to the humoral immune responses that are mutually complementary with cellular immunity [52]. The facilitated differentiation of Th cells into distinct subsets was found to be related as well, especially Th1 and Th17 subsets that play important roles in tumor therapy by coordinating cytotoxic T cell and NK cell activity [53]. Collectively, this retrieved information from transcriptome sequencing analysis further enhanced the understanding of the modulation of the immune landscape by Se‐*Ssp*.

**FIGURE 5:**
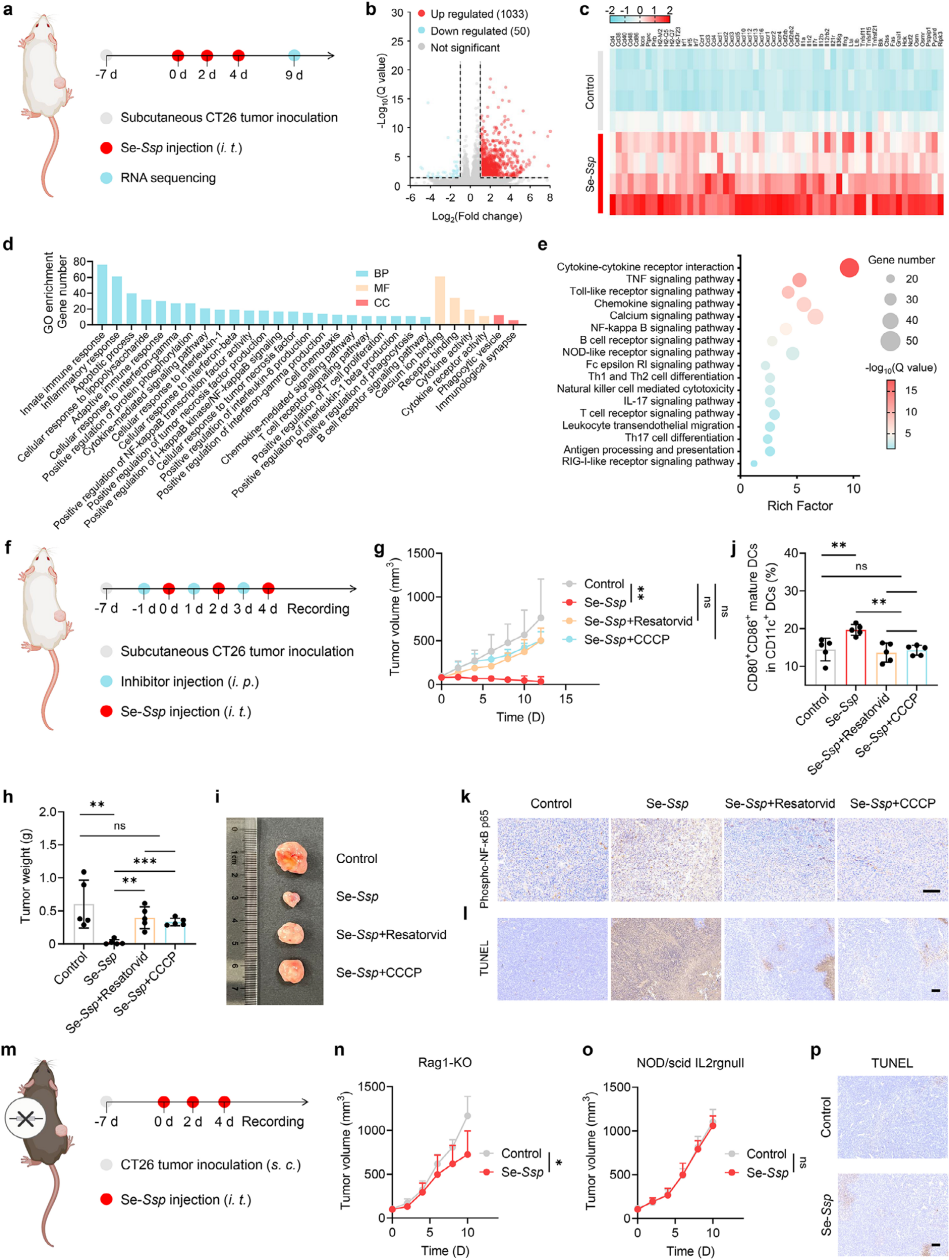
Therapeutic mechanisms of the intratumoral injection of Se‐*Ssp* in the CT26 subcutaneous tumor model. (a) Schematic illustration and timeline of Se‐*Ssp* intratumoral injection and RNA‐seq in the CT26 colon tumors from Balb/c mice. (b‐e) Transcriptome high‐throughput sequencing of the control and Se‐*Ssp* groups (n = 4, q < 0.05, fold change > 2). (b) Volcano plot of identified DEGs in the control and Se‐*Ssp* groups. (c) DEG heatmap of the control and Se‐*Ssp* groups. d‐e, (d) GO enrichment and (e) KEGG enrichment of DEG analysis in the control and Se‐*Ssp* groups. (f) Schematic illustration and timeline of Se‐*Ssp* intratumoral injection with inhibitors in the CT26 colon tumors on Balb/c mice. (g‐k) Groups: control (n = 5), Se‐*Ssp* (n = 5), Se‐*Ssp*+Resatorvid (n = 5), and Se‐*Ssp*+CCCP (n = 5). (g) The average tumor volume curves. (h) The average tumor weights. (i) Representative tumor photograph. (j) The quantification of DC maturation in TDLNs. (k, l) Representative immunohistochemical images of (k) Phospho‐NF‐κB p65 and (l) TUNEL of tumor sections from Balb/c mice. (m‐p), (m) Schematic illustration and timeline of Se‐*Ssp* intratumoral injection in the CT26 colon tumors on Rag1‐KO mice and NSG mice. (n) The average tumor volume curves on Rag1‐KO mice, groups: control (n = 4) and Se‐*Ssp* (n = 4). (o) The average tumor volume curves on NSG mice, groups: control (n = 5) and Se‐*Ssp* (n = 5). (p) Representative immunohistochemical images of TUNEL of tumor sections from NSG mice. Scale bar: 100 µm. Data are presented as the mean ± SD. Statistical significance was calculated via unpaired two‐tailed Student's t‐test and expressed as ns *p* > 0.05, *p < 0.05, **p < 0.01, and ***p < 0.001.

To elucidate the roles of the TLR and cGAS‐STING pathways in initiating immune activation in the treatment of Se‐*Ssp*, Resatorvid and CCCP were administered to mice via the intraperitoneal route (Figure 5f). According to the tumor growth curves, these two additional inhibitors significantly attenuated the therapeutic effects of Se‐*Ssp* (Figure 5g, Figure S6a). On day 12, while the tumors in the Se‐*Ssp*‐only group regressed, there were only slight differences in tumor volume and weight among the control, Se‐*Ssp*+Resatorvid, and Se‐*Ssp*+CCCP groups (Figure 5h,i, Figure S6b,e). Additionally, IHC analysis revealed that the area positive for phospho‐NF‐κB decreased, indicating that blockade of the TLR and cGAS‐STING signaling networks led to impaired activation of the downstream NK‐κB pathway (Figure 5k). Consequently, the maturation levels of DCs in both TDLNs and tumors were decreased in the mice that were administered these two inhibitors (Figure 5j, Figure S6c). This inhibition of innate immunity weakened T‐cell infiltration around the tumor sites (Figure S6d). Based on this, the subsequent adaptive immune effects were negatively influenced, as evidenced by a remarkable reduction in TUNEL‐positive apoptotic cells in the tumor tissues of the Resatorvid and CCCP groups (Figure 5l). These findings underscored that Se‐*Ssp* was involved in the TLR and cGAS‐STING pathways for oncotherapy, activating innate and subsequent adaptive immunity.

Next, the immune‐deficient mouse models were employed to further confirm the roles of immune responses in tumor eradication (Figure 5m). Rag1‐knockout (Rag1‐KO) mice are genetically engineered mice lacking recombination‐activating gene 1 (Rag1), which is essential for the development of T lymphocytes. In fact, only modest therapeutic effects were observed when Rag1‐KO mice were subjected to Se‐*Ssp*, as indicated by the tumor volumes, tumor weights, and TUNEL staining of tumor sections (Figure 5n, Figure S7a‐c,e,i). Considering the dysfunction of T cells, this limited response was likely attributed to the initiated immunity mediated by Se‐*Ssp*, since it could still trigger DC maturation (Figure S7d). From another perspective, these results also highlighted the indispensability of adaptive immunity for Se‐*Ssp*‐mediated cancer immunotherapy. Additionally, Se‐*Ssp* exhibited negligible activity in severely immunodeficient NOD/scid IL2‐null (NSG) mice (Figure 5o). The tumor volume, weight, and TUNEL staining results did not display significant differences between the untreated and treated mice, indicating that the therapeutic outcomes relied heavily on the immunostimulatory effects of Se‐*Ssp* (Figure 5p, Figure S7f‐h,j).

### 2.7 Potential Clinical Application of Se‐*Ssp*

The natural fabrication process of Se NPs on *Ssp* enabled the potential large‐scale production of Se‐*Ssp*, which meant that these engineered microalgae featured good compatibility with promising clinical application potential. As indicated previously, although the percentage of Tregs in the Se‐*Ssp* group was lower than that in the *Ssp* group, it remained higher than that in the control group. These findings indicated that Se‐*Ssp* could be combined with several clinical agents that target Tregs to promote their efficacy. For example, given that Tregs are known to suppress CTL activity, we hypothesized that Se‐*Ssp* could simultaneously modulate immunosuppressive Tregs by increasing the sensitivity of the host to αCTLA‐4 antibody (Figure 6a) [54]. Herein, bilateral CT26‐bearing mice were divided into four groups, including control, Se‐*Ssp*, αCTLA‐4, and Se‐*Ssp*+αCTLA‐4. The tumors on the right side were designated as “primary tumors” for localized therapy with three injections of Se‐*Ssp*, while the tumors on the left side were designated as “distant tumors” without any direct treatment. The Se‐*Ssp* performed well on unilateral tumors, as demonstrated previously; however, its performance in the bilateral model was less than ideal, with a marginal suppression of the tumor volume of distant tumors and a survival rate of 1/5. As for ICB alone, intervention with αCTLA‐4 antibody had little effect on combating tumors in this setting. Strikingly, upon co‐administration of the two substances, the combined therapeutic outcomes were improved for not only primary but also distant tumors, which ultimately improved the survival rate to 9/10 (Figure 6b‐e, Figure S8a,b). Moreover, the CD8 signal was also enhanced in both primary and distant tumor slices, suggesting that the combination strategy might directly attenuate the immunosuppressive capacity of Tregs while blocking inhibitory signals on CD8^+^ CTLs, thereby remodeling a beneficial TME for more efficient and longer‐term anti‐tumor immunity (Figure 6f). In addition to the αCTLA‐4 antibody commonly used in clinical practice, NLG919 is a small‐molecule immunomodulatory agent that acts as an IDO1 inhibitor [55]. However, NLG919 ultimately failed to achieve the expected efficacy in clinical trials owing to its insufficient efficacy [56]. We then combined Se‐*Ssp* with NLG919 in bilateral CT26‐bearing mice to investigate whether Se‐*Ssp* could increase the responsiveness of the body to NLG919 to overcome its clinical limitations (Figure 6g). As expected, the primary and distant tumors treated with Se‐*Ssp* and NLG919 concomitantly were suppressed more than those treated with NLG919 monotherapy (Figure 6h,l, Figure S8c‐f). Thus, Se‐*Ssp* revealed the potential support to rescue the clinical re‐development of NLG919 in some respects.

**FIGURE 6:**
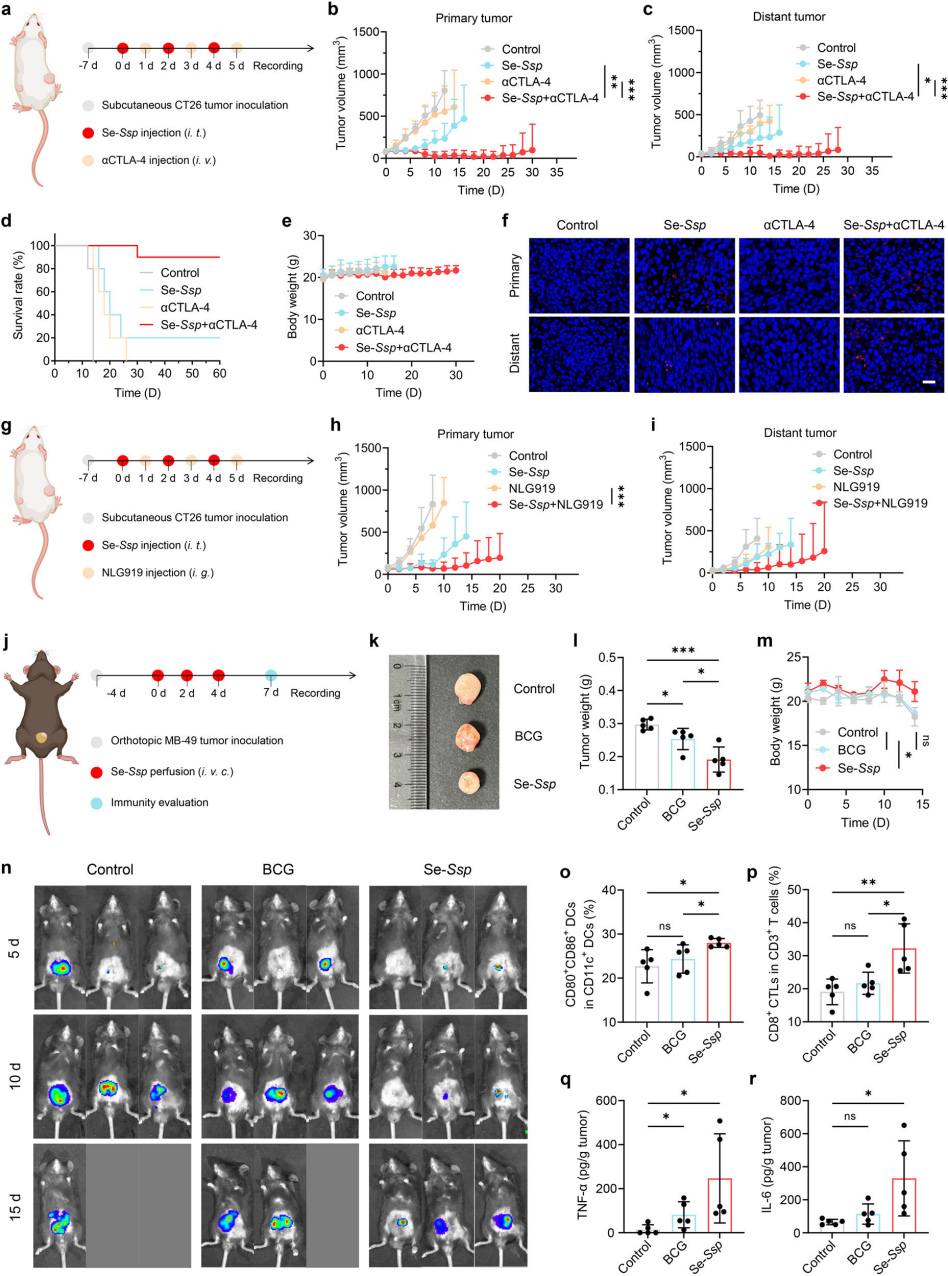
The potential clinical application of Se‐*Ssp* in the CT26 subcutaneous tumor and MB‐49 orthotopic bladder tumor models. (a) Schematic illustration and timeline of Se‐*Ssp* intratumoral injection combined with αCTLA‐4 in the CT26 colon tumors on Balb/c mice. (b‐f) Groups: control (n = 5), Se‐*Ssp* (n = 5), αCTLA‐4 (n = 5), and Se‐*Ssp*+αCTLA‐4 (n = 10). b‐c, The average (b) primary tumor and (c) distant tumor volume curves. (d) The overall survival rates. (e) The average body weight. (f) Representative immunofluorescence images of CD8 in primary and distant tumor sections. (g) Schematic illustration and timeline of Se‐*Ssp* intratumoral injection combined with NLG919 in the CT26 colon tumors on Balb/c mice. (h‐i) Groups: Groups: control (n = 5), Se‐*Ssp* (n = 5), NLG919 (n = 5), and Se‐*Ssp*+NLG919 (n = 5). The average (h) primary tumor and (i) distant tumor volume curves. (j) Schematic illustration and timeline of Se‐*Ssp* instillation in the orthotopic MB‐49 bladder tumor on C57/BL6 mice. (k, l) Groups: control (n = 5), BCG (n = 5), and Se‐*Ssp* (n = 5). (k) Representative tumor photograph. (l) The average tumor weights. m‐n, Groups: Control (n = 3), BCG (n = 3), and Se‐*Ssp* (n = 3). (m) The average body weights. (n) Representative bioluminescence images of individual mice on day 5, day 10, and day 15. (o‐r) Groups: control (n = 5), BCG (n = 5), and Se‐*Ssp* (n = 5). (o‐p) The quantification of (o) DC maturation and (p) the percentages of CTLs in bladder tumors. (q‐r) the ELISA results for (q) TNF‐α and (r) IL‐6 in bladder tumors. Scale bar: 20 µm. Data are presented as the mean ± SD. Statistical significance was calculated via unpaired two‐tailed Student's t‐test and expressed as ns *p* > 0.05, *p < 0.05, **p < 0.01, and ***p < 0.001.

Despite achieving favorable therapeutic outcomes, intratumoral injection remains restricted in clinical applicability. On the other hand, microbial therapy is particularly well‐suited for bladder perfusion. The microscale size of microorganisms favors localized delivery rather than systemic administration, while the bladder provides a confined and accessible space to improve efficiency and avoid systemic infections [14, 57]. Prime examples such as the BCG vaccine can directly interact with bladder tumor cells, eliciting strong local immune responses. Therefore, we considered the application of Se‐*Ssp* in bladder cancer immunotherapy (Figure 6j). Mice with orthotopic MB‐49 or MB‐49‐luc tumors were randomly assigned to three groups, namely, the control, BCG (5 mg), and Se‐*Ssp* (2 × 10^7^ cells) groups [14, 57]. The Se‐*Ssp*‐treated group exhibited the weakest bioluminescence signal, which was even lower than that in the BCG, the clinically standard group (Figure 6n). Relatively comprehensive evaluations, including photographs, B‐mode ultrasound scans, and weights of bladder tumors collected on day 7 after the first instillation, consistently indicated that Se‐*Ssp* could suppress orthotopic tumors to a certain extent (Figure 6k,l, Figure S9a,b). H&E staining revealed a necrotic area in the sections of the tumors treated with Se‐*Ssp* (Figure S9e). Throughout the monitoring period, the mice in both the control and BCG vaccine groups exhibited progressive weight loss, with a more pronounced reduction observed at later time points. Fortunately, mice treated with Se‐*Ssp* presented a relatively gradual decline in body weight, indirectly reflecting that Se‐*Ssp* delayed bladder tumor development (Figure 6m). Immune profiling of the whole bladder was further conducted. Compared with the BCG vaccine, Se‐*Ssp* resulted in higher stimulation of DC maturation, thereby significantly promoting CTL and Th cell infiltrations (Figure 6o,p, Figure S9c,f). Notably, the population of Tregs in bladder tumors was also reduced by Se‐*Ssp* (Figure S9d). The supernatants of tumors in the Se‐*Ssp* groups contained relatively high concentrations of pro‐inflammatory cytokines, including at least TNF‐α and IL‐6 (Figure 6q‐r). Meanwhile, H&E staining did not reveal any significant inflammatory injury to the urothelium, lamina propria, or muscularis propria of the bladder after perfusion with Se‐*Ssp* (Figure S9g). Taken together, these findings illustrated that Se‐*Ssp* had promising application prospects in combating bladder tumors via perfusion, the efficacy of which even surpassed that of standard BCG therapy in some situations.

## 3 Conclusions and Perspectives

In summary, we developed Se‐enriched microalgae by exposing *Ssp* to Na_2_SeO_3_. The obtained Se‐*Ssp* with good biocompatibility could serve as a potent immune modulator to boost DC maturation via at least the TLR and Se‐enhanced cGAS‐STING pathways. As evidenced by the mouse CT26 colon tumor model, intratumoral injections of Se‐*Ssp* could trigger robust innate immunity, followed by the enhancement of adaptive immunity; these immunostimulatory effects led to impressive anti‐tumor potency with long‐term immune memory. Additionally, Se‐*Ssp* magnified the sensitivity of the body to clinical ICI, αCTLA‐4 antibody, and the IDO small‐molecule inhibitor NLG919, since the combinations suppressed both primary and distant tumor progression. In addition, local perfusion administration of Se‐*Ssp* into tumors is a realistic way to achieve translation by increasing Se‐*Ssp* efficiency while decreasing systemic toxicity and cytokine storms; compared with the standard clinical BCG vaccine, Se‐*Ssp* displayed better immune activation and tumor inhibition effects. Overall, this study successfully established a potent microorganism‐based immunotherapy by utilizing naturally engineered Se‐*Ssp*, elucidating its underlying mechanisms of immunomodulation. Meanwhile, it was demonstrated the translational potential of this kind of engineered microalgae, particularly as ICI or IDO inhibitor sensitizers and as a candidate agent for bladder instillation. These findings provided a novel platform for oncology treatment, with the promise to enhance patient care. However, compared with small molecules or antibodies, microorganism‐based immune agents still face additional challenges. Future strategies may include attenuation or genetic modification to reduce potential risks, encapsulation or carrier‐based delivery to enhance stability and bioavailability, and lyophilization to improve shelf‐life. In addition, further validation in more physiologically relevant models, such as orthotopic tumor models, will be essential to better assess their therapeutic efficacy and safety, thereby promoting their clinical transformation.

## Author Contributions

L.C. and Y.C. oversaw all research. Y.Y., X.Z., and L.C. designed the experiments. Y.Y., X.Z., and J.Q. performed the fabrication and characterization. Y.Y. and T.Q. performed the cell experiments. Y.Y., X.C., and Q.L. performed animal experiments. Y.Y., X.Z., Y.C., and L.C. wrote and revised the manuscript. All authors reviewed and edited the manuscript.

## Conflicts of Interest

The authors declare no conflicts of interest.

## Supporting information




**Supporting File**: advs77297‐sup‐0001‐SuppMat.docx.

## Data Availability

The data that support the findings of this study are available from the corresponding author upon reasonable request.
