# Supplementary material for: Natural‐Inspired Engineered Microalgae With Selenium Enrichment Enabled Potent Anti‐Tumor Immunity
Source: Adv Sci (Weinh). 2026 Aug 27:e77297. Online ahead of print. doi: 10.1002/advs.77297 (PMC13521181; doi:10.1002/advs.77297)
Supplement: Supplementary file 1 [file ADVS-9999-e77297-s001.docx]

**Supporting Information**

**Natural-Inspired Engineered Microalgae with Selenium Enrichment Enabled Potent Anti-tumor Immunity**

*Yuqi Yang^[a,b,c]^, Xiaowei Cai^[d]^, Xiaoyan Zhong^[a,e]^*, Tian Qiu^[a,e]^, Qinwei Lu^[a]^, Jiahao Qin^[a,b,c]^,*

*Fei Gong^[a]^, Yecheng Huang^[a]^, Chenyu Jiang^[a]^, Youdong Chen^[a]^, Yuan Cheng^[b,c]^*,*

*and Liang Cheng^[a]^**

[a] Miss Y. Yang, Dr. X. Zhong, Miss T. Qiu, Dr. Q. Lu, Mr. J. Qin, Dr. F. Gong, Miss Y. Huang, Dr. C. Jiang, Mr. Y. Chen, Prof. L. Cheng

Institute of Functional Nano & Soft Materials (FUNSOM),

Jiangsu Key Laboratory of Advanced Healthcare Materials & Devices,

Biomedical-BasicResearch-Center (BBRC) of Jiangsu Province,

Soochow University, Suzhou, 215123, China

E-mail: [lcheng2@suda.edu.cn](mailto:lcheng2@suda.edu.cn); [xyzhong@suda.edu.cn](mailto:xyzhong@suda.edu.cn)

[b] Miss Y. Yang, Mr. J. Qin, Prof. Y. Cheng

Suzhou Industrial Park Monash Research Institute of Science and Technology,

Monash University, Suzhou, 215000, China

[c] Miss Y. Yang, Mr. J. Qin, Prof. Y. Cheng

Department of Materials Science and Engineering, Faculty of Engineering

Monash University, Clayton, VIC3800, Australia

E-mail: yuan.cheng@monash.edu

[d] Mr. X. Cai

Department of Urology,

The Second Affiliated Hospital of Soochow University, Suzhou, 215004, China

[e] Dr. X. Zhong, Miss T. Qiu

Department of Toxicology, School of Public Health,

Suzhou Medical College of Soochow University, Suzhou, 215123, China

1. **Methodology**

***Microalgae culture and Se enrichment***

The microalgae *Synechococcus. sp* (FACHB-805 strain, labelled as *Ssp*) was purchased from the Freshwater Algae Culture Collection at the Institute of Hydrobiology, China. *Ssp* was cultured in blue-green medium 11 (BG11) medium (Hopebiol, China) in an Erlenmeyer flask under constant 25 °C with white light (6000 lux) for 12 h per day; once its growth reached the logarithmic phase, follow-up procedures were carried out.

To engineer Se-enriched *Ssp* (Se-*Ssp*), 10^7^ /mL *Ssp* was incubated in BG11 medium containing 10 mM Na_2_SeO_3_, under constant 25 °C with white light (6000 lux) for 12 h per day. On day 6, the Se-*Ssp* were collected and washed for further use.

***Se nanoparticles synthesis and purification***

To purify Se nanoparticles (Se NPs), the sonicated Se-*Ssp* suspensions were collected and centrifuged first. The resulting supernatants were then filtered through a 0.45 μm membrane filter. The collected Se NPs were then washed with ddH_2_O and stored at 4 °C for further use.

To simulate the biosynthesis of Se NPs by *Ssp*, 100 μM ascorbic acid or 5 mM glutathione was added to 10 mM Na_2_SeO_3_ and stirred for 1 h at 25 °C. The collected Se NPs from GSH (G-Se NPs) were then washed with ddH_2_O and stored at 4 °C for further use.

***Characterization***

The morphology of *Ssp* and Se-*Ssp* was observed by scanning electron microscopy (SEM, Zeiss G500) and transmission electron microscopy (TEM, Tecnai FEI TF20). The element mapping of Se NPs was characterized by another TEM (Thermo Scientific TALOS 200X). The hydrodynamic size and zeta potential were tested by a Zeta sizer (Malvern Nano ZS90). UV-vis-NIR absorbance spectra were all recorded by a spectrophotometer (Thermo Scientific GNESYS 50). The Se concentrations of Se-*Ssp* and Se NPs were determined by an inductively coupled plasma optical emission spectrometry (ICP-OES, PerkinElmer Avio 200). The valence of Se from Se NPs was measured by X-ray photoelectron spectroscopy (XPS, Thermo ESCAlab 250Xi). The sonicated *Ssp* and Se-*Ssp* suspensions, and Se NPs were treated according to the recommended protocol and then run on a 4~20% SDS-PAGE gel with a prestained protein ladder (Sparkjade, China) for subsequent Coomassie Brilliant Blue G-250 staining (KeyGEN, China).

***Cell culture***

The murine colorectal cancer cell line CT26 and the murine mammary cancer cell line 4T1, and the human umbilical vein endothelial cells (HUVECs) were purchased from the Cell Bank, Shanghai Institutes for Biological Sciences, Chinese Academy of Sciences. The murine prostate cancer cell line RM-1, the murine bone marrow-derived dendritic cell line DC2.4, and the murine macrophage line RAW 264.7 were purchased from Wuhan Pricella Biotechnology. The murine bladder cancer cell lines MB-49 and MB-49 luc were a gift from Z. Liu at Soochow University. CT26, 4T1, RM-1, DC2.4, MB-49, and MB-49-luc were cultured in RPMI-1640 complete medium in a standard cell culture environment (37 ℃, 5% CO_2_). HUVECs and RAW 264.7 and were cultured in DMEM complete medium in a standard cell culture environment (37 °C, 5% CO_2_).

***Biocompatibility***

To determine *in vitro* cell viability, the standard 3-(4,5-dimethyl-2-thiazolyl)-2,5-diphenyl-2-tetrazolium bromide (MTT) assay was performed to determine the relative cell viability after incubation with Se-*Ssp* for 12 h; the absorption was recorded by a microplate reader (Biotek Synergy H1).

To test the cell apoptosis rate, the DC2.4 cells were incubated with Se-*Ssp* for 12 h, and then were stained with Annexin V-FITC and PI (MeilunBio, China) according to the recommended protocol. The collected cells were analyzed by flow cytometry (BD Accuri C6 Plus).

***Endocytosis***

To observe endocytosis, the DC2.4 cells were incubated with *Ssp* and Se-*Ssp* labelled with FITC by using FITC-NHS (Stragraydye, China) via amide bond reaction. After 6 h and 12 h, the collected cells were photographed by a confocal laser scanning microscope (CLSM) and analyzed by flow cytometry, respectively.

***DC maturation***

Bone marrow-derived dendritic cells (BMDCs) were obtained from the bone marrow of female mice (C57BL/6, 6~8 weeks old) and then cultured with granulocyte macrophage-colony stimulating factor (GM-CSF, Peprotech, USA) according to the recommended protocol. With a maturation level (percentage of CD80^+^CD86^+^ in CD11c^+^ DCs) around 15%~25%, these BMDCs on days 5~7 were collected for further studies.

BMDCs were incubated with 500 ng/mL LPS (Biosharp, China), 5×10^6^ /mL *Ssp* and 5×10^6^ /mL Se-*Ssp* for 12 h, followed by being collected and blocked with anti-mouse CD16/32 antibody. For DC maturation, these BMDCs were stained with FITC anti-mouse CD11c, PE anti-mouse CD80, and PE-Cy7 anti-mouse CD86 before analysis by flow cytometry (Fongcyte); mature DCs were recognized as CD11c^+^CD80^+^CD86^+^. For CD40 expression, these BMDCs were stained with FITC anti-mouse CD11c and PE anti-mouse CD40 (Biolegend, USA) before analysis by flow cytometry (Challenbio Fongcyte). For antigen presentation, these BMDCs were stained with FITC anti-mouse CD11c and PE anti-mouse MHC II; antigen-presenting DCs were recognized as CD11c^+^MHC II^high^. The supernatants of these BMDCs were also collected to measure the levels of cytokines by ELISA kits, including tumor necrosis factor α (TNF-α, Invitrogen, USA), interleukin 6 (IL-6, Invitrogen, USA), and interleukin 1β (IL-1β, Finetest, China). BMDCs were incubated with the supernatants of LPS, *Ssp* and Se-*Ssp* for 12 h by using a 0.4 μm transwell (Nest Biotechnology, China), and analyzed following the same protocol.

RAW264.7 were incubated with 500 ng/mL LPS (Biosharp, China), 5×10^6^ /mL *Ssp* and 5×10^6^ /mL Se-*Ssp* for 12 h, followed by being collected, blocked with anti-mouse CD16/32 antibody. For M1-phenotype polarization, these RAW264.7 cells were stained with FITC anti-mouse F4/80, PE anti-mouse-CD80 before analysis by flow cytometry (Fongcyte); M1-phenotype macrophages were F4/80^+^CD80^+^. For M2-phenotype polarization, these RAW264.7 cells were stained with FITC anti-mouse F4/80, PE anti-mouse-CD206 before analysis by flow cytometry (Fongcyte); M2-phenotype macrophages were F4/80^+^CD206^+^.

***ROS measurement***

To measure the ROS level, DC2.4 cells were incubated with 5×10^6^ /mL *Ssp*, 5 μg/mL Se NPs, and 5×10^6^ /mL Se-*Ssp* for 12 h, followed by being incubated with dichlorodihydrofluorescein diacetate (DCFH-DA, 20 μM) for 1 h to capture intracellular ROS by flow cytometry (Fongcyte).

***TLR pathway***

To demonstrate TLR4 pathway activation, BMDCs were incubated with 100 nM Resatorvid (MedChemExpress, China) for 4 h, then 5×10^6^ /mL *Ssp*, 5 μg/mL Se NPs, and 5×10^6^ /mL Se-*Ssp* for 12 h, followed by being collected and blocked with anti-mouse CD16/32 antibody. For DC maturation, these BMDCs were stained with FITC anti-mouse CD11c, PE anti-mouse CD80, and PE-Cy7 anti-mouse CD86 before analysis by flow cytometry (Fongcyte); mature DCs were recognized as CD11c^+^CD80^+^CD86^+^.

In addition, HEK-Dual mTLR4 (NF/IL8) cells (Invivogen, USA) were incubated with Se NPs, the supernatants of *Ssp* and Se-*Ssp* for 12 h by using a 0.4 μm transwell (Nest Biotechnology, China), with LPS serving as a positive control. After removing the transwell, the supernatants of HEK-Dual mTLR4 (NF/IL8) cells in the lower chamber were collected, lysed, and incubated with coelenterazine (Invivogen, USA) according to the recommended protocol, followed by bioluminescence intensity recording using a microplate reader (Thermo Scientific Variskan).

***STING pathway***

To demonstrate STING pathway activation, BMDCs were incubated with 10 μM carbonyl cyanide 3-chlorophenylhydrazone (CCCP, Solarbio, China) for 4 h, then 5×10^6^ /mL *Ssp*, 5 μg/mL Se NPs, and 5×10^6^ /mL Se-*Ssp* for 12 h, followed by being collected and blocked with anti-mouse CD16/32 antibody. These BMDCs were stained with FITC anti-mouse CD11c, PE anti-mouse CD80, and PE-Cy7 anti-mouse CD86 before analysis by flow cytometry (Fongcyte); mature DCs were recognized as CD11c^+^CD80^+^CD86^+^. The supernatants of these BMDCs were also collected to measure the levels of interferon β (IFN-β) by ELISA kits (R&D System, USA).

Western blotting analysis was conducted to investigate the activation of the STING pathway. DC2.4 cells were incubated with 5×10^6^ /mL *Ssp*, 5 μg/mL Se NPs, and 5×10^6^ /mL Se-*Ssp* for 12 h, followed by being collected and lysed for protein extraction. The protein concentration was quantified using the bicinchoninic acid (BCA) method. Then, western blotting was performed to determine the expression of proteins related to the STING pathway, including STING/Phospho-STING, TBK-1/NAK/Phospho-TBK-1/NAK, IRF-3/Phospho-IRF-3.

***Se-induced Ca ion pathway***

BMDCs were incubated with Na_2_SeO_3_, G-Se NPs, Se NPs, and Se-*Ssp* containing 5 μg/mL Se, followed by being collected and blocked with anti-mouse CD16/32 antibody. For DC maturation, these BMDCs were stained with FITC anti-mouse CD11c, PE anti-mouse CD80, and PE-Cy7 anti-mouse CD86 before analysis by flow cytometry; mature DCs were recognized as CD11c^+^CD80^+^CD86^+^.

Western blotting analysis was conducted to investigate the expression of selenoprotein K (SELENOK). DC2.4 cells were incubated with 5×10^6^ /mL *Ssp*, 5 μg/mL Se NPs, and 5×10^6^ /mL Se-*Ssp* for 12 h, followed by being collected and lysed for protein extraction. The protein concentration was quantified using the bicinchoninic acid (BCA, Beyotime, China) kit. Then, western blotting was performed to determine the expression of SELENOK.

To test the enhanced Ca signal in cytoplasm, DC2.4 cells were incubated with 5×10^6^ /mL *Ssp*, 5 μg/mL Se NPs, and 5×10^6^ /mL Se-*Ssp* for 12 h, followed by being collected and stained with 4 μM Fluo-4 AM (Maokangbio, China); the signal of Fluo-4 AM was determined by flow cytometry.

To demonstrate Ca ion pathway activation, BMDCs were incubated with 1 μM Nifedipine (AbMole, USA) for 1 h, then 5×10^6^ /mL *Ssp*, 5 μg/mL Se NPs, and 5×10^6^ /mL Se-*Ssp* for 12 h, followed by being collected and blocked with anti-CD16/32 antibody. These BMDCs were stained with FITC anti-mouse CD11c, PE anti-mouse CD80, and PE-Cy7 anti-mouse CD86 before analysis by flow cytometry (Fongcyte); mature DCs were recognized as CD11c^+^CD80^+^CD86^+^.

***NF-κB pathway***

Western blotting analysis was conducted to investigate the activation of the NF-κB pathway. DC2.4 cells were incubated with 5×10^6^ /mL *Ssp*, 5 μg/mL Se NPs, and 5×10^6^ /mL Se-*Ssp* for 12 h, followed by being collected and lysed for protein extraction. The protein concentration was quantified using the BCA kit. Then, western blotting was performed to determine the expression of IκBα/Phospho-IκBα and NF-κB p65/Phospho-NF-κB p65.

To observe the activation of nuclear translocation of Phospho-NF-κB p65, immunofluorescence was conducted. DC2.4 cells were incubated with 5×10^6^ /mL *Ssp*, 5 μg/mL Se NPs, and 5×10^6^ /mL Se-*Ssp* for 12 h, followed by being stained with NF-κB p65 and Phospho-NF-κB p65 as the primary antibodies, and Alexa Fluor™ 647 (Proteintech, China) as the secondary antibody; the collected cells were photographed by CLSM.

***Animal tumor model***

The animal experiments were conducted under protocols approved by the Soochow University Laboratory Animal Center (No. ECSU-202504A0009). Female Balb/c mice (6~8 weeks old) and NOD/scid IL2rgnull mice (6~8 weeks old), male C57BL/6 mice (6~8 weeks old), and Rag1-KO were purchased from Hangzhou Enlighten the Truth Laboratory Animal Technology Co., Ltd. and chosen for the experimental tumor model.

***Immune evaluation***

For the immune evaluation of the CT26 tumor model, 50 μL of PBS containing 2×10^6^ CT26 cells was subcutaneously injected into the right leg of the female Balb/c mice. The CT26 tumor-bearing mice were randomly divided into four groups: (1) control; (2) *Ssp* (*i. t.*, 2×10^8^ /mL, 50 μL); (3) Se NPs (*i. t.*, 200 μg/mL, 50 μL); and (4) Se-*Ssp* (*i. t.*, 2×10^8^ /mL, 50 μL). The injections were repeated three times. CT26 tumors and tumor-draining lymph nodes (n = 5 per group) were collected from mice on day 3 and day 7 after the last treatment, followed by homogenization into single-cell suspensions according to the recommended protocol. Then, these cell suspensions were stained with different antibodies and subjected to flow cytometry analysis. All cell samples were first blocked with anti-mouse CD16/32 antibody. DCs in the lymph nodes were stained with FITC anti-mouse CD11c, PE anti-mouse MHC II, APC anti-mouse CD80, and PE-Cy7 anti-mouse CD86; mature DCs were recognized by CD11c^+^CD80^+^CD86^+^, resident mature DCs were recognized by CD11c^+^MHC II^low^CD80^+^CD86^+^, and migrant mature DCs were recognized by CD11c^+^MHC II^high^CD80^+^CD86^+^. Considering the influence of residual phycocyanin from *Ssp*, APC-conjugated antibodies were not used for the cells in the tumor. DCs in the tumor were stained with FITC anti-mouse CD11c, PerCP anti-mouse CD45, PE-Cy7 anti-mouse CD80; mature DCs were recognized by CD45^+^CD11c^+^CD80^+^. PD-L1-expressing DCs were stained with FITC anti-mouse CD11c, PE anti-mouse CD274, PE-Cy7 anti-mouse CD45; PD-L1-expressing DCs were recognized by CD45^+^CD11c^+^CD274^+^. M1-phynotype macrophages in the tumor were stained with FITC anti-mouse F4/80, PE anti-mouse CD11b, PerCP anti-mouse CD45, and PE-Cy7 anti-mouse CD80; M1-phynotype macrophages were recognized by CD45^+^CD11b^+^F4/80^+^CD80^+^. M2-phynotype macrophages in the tumor were stained with FITC anti-mouse F4/80, PE anti-mouse CD11b, PerCP anti-mouse CD45, and PE-Cy7 anti-mouse CD206; M2-phynotype macrophages were recognized by CD45^+^CD11b^+^F4/80^+^CD206^+^. T cells in the tumor were stained with FITC anti-mouse CD3, PE anti-mouse CD8a, PerCP anti-mouse CD45, and PE-Cy7 anti-mouse CD4; CTLs were recognized by CD45^+^CD3^+^CD8a^+^, and Th cells were recognized by CD45^+^CD3^+^CD4^+^. Gzmb-expressing CTLs were stained with FITC anti-mouse Gzmb, PE anti-mouse CD3, PerCP anti-mouse CD8, and PE-Cy7 anti-mouse CD45. Tregs in the tumor were stained with FITC anti-mouse CD3, PE anti-mouse FOXP3, PerCP anti-mouse CD45, and PE-Cy7 anti-mouse CD4; Tregs were recognized by CD45^+^CD3^+^CD4^+^FOXP3^+^. The supernatants from the tumor tissues were collected to measure the relative levels of cytokines via ELISA kits, including TNF-α (Invitrogen, USA), IFN-β (R&D System, USA), IFN-γ (Invitrogen, USA), IL-6 (Invitrogen, USA), and IL-10 (Invitrogen, USA).

***Therapeutical outcomes***

For the hemolysis assay, freshly isolated red blood cells (RBCs) collected from healthy mice were diluted into a 4% RBC suspension, followed by incubation with Se-*Ssp* at different concentrations at 37 °C for 1 h. 0.9% saline solution and ddH_2_O were used as negative and positive controls, respectively. After incubation, the mixtures were centrifuged at 5000 rpm for 5 min to collect the supernatant for measuring the absorbance at 544 nm by a microplate reader (Biotek Synergy H1). The hemolysis rate was calculated by the formula Hemolysis (%) = (Absorbance _Sample_ - Absorbance _Saline_)/ (Absorbance _ddH2O_ - Absorbance _Saline_).

For the therapeutic assessment of the CT26 tumor model, 50 μL of PBS containing 2×10^6^ CT26 cells was subcutaneously injected into the right leg of the female Balb/c mice. The CT26 tumor-bearing mice were randomly divided into four groups: (1) control; (2) *Ssp* (*i. t.*, 2×10^8^ /mL, 50 μL); (3) Se NPs (*i. t.*, 200 μg/mL, 50 μL); and (4) *Se-Ssp* (*i. t.*, 2×10^8^ /mL, 50 μL). The tumor length and width were recorded 2 every two days to calculate the tumor volume by the formula Volume = Length × Width^2^/2. The tumors were subjected to hematoxylin and eosin (H&E), TUNEL, and Ki67 immunohistochemistry staining.

***Immune memory***

For tumor rechallenge, 50 μL of PBS containing 2×10^6^ CT26 cells was subcutaneously injected into the left leg of the Balb/c mice after 60 days of the treatment. Healthy mice were used as the naive control. The tumor length and width were recorded every two days to calculate the tumor volume by the formula Volume = Length × Width^2^/2. On day 59, peripheral blood from tumor-rechallenged mice was collected to lyse the erythrocytes and leave the leukocytes. The remaining cells were blocked by anti-mouse CD16/32 antibody at first, followed by being stained with FITC anti-mouse CD44, PE anti-mouse CD8a, APC anti-mouse CD62L, PerCP anti-mouse CD3, PE-Cy7 anti-mouse CD4; CD8^+^ Tem cells were recognized by CD3^+^CD8^+^CD44^high^CD62L^low^, CD8^+^ Tcm were recognized by CD3^+^CD8^+^CD44^high^CD62L^high^, CD4^+^ Tem cells were recognized by CD3^+^CD4^+^CD44^high^CD62L^low^, and CD4^+^ Tcm were recognized by CD3^+^CD4^+^CD44^high^CD62L^high^.

***In vivo mechanism***

For transcriptome sequencing, 50 μL of PBS containing 2×10^6^ CT26 cells was subcutaneously injected into the right leg of the female Balb/c mice. The CT26 tumor-bearing mice were divided randomly into two groups: (1) control; (2) Se-*Ssp* (*i. t.*, 2×10^8^ /mL, 50 μL). The tumors that were subjected to transcriptome sequencing were collected on day 5 after the last treatment.

For blocking relative pathway therapy, the CT26 tumor-bearing mice were divided randomly into four groups: (1) control; (2) Se-*Ssp* (*i. t.*, 2×10^8^ /mL, 50 μL); (3) Se-*Ssp* (*i. t.*, 2×10^8^ /mL, 50 μL) + Resatorvid (*i.p.*, 2 mg/kg for three times); (4) Se-*Ssp* (*i. t.*, 2×10^8^ /mL, 50 μL) + CCCP (*i.p.*, 2 mg/kg for three times). Resatorvid and CCCP were injected on -1 d, 1 d, and 3 d. The tumor length and width were recorded every two days to calculate the tumor volume by the formula Volume = Length × Width^2^/2. The lymph nodes and tumors were collected on day 12, followed by homogenization into single-cell suspensions according to the recommended protocol. Then, these cell suspensions were stained with different antibodies and subjected to flow cytometry analysis. All cell samples were first blocked with anti-mouse CD16/32 antibody. DCs in the lymph nodes were stained with FITC anti-mouse CD11c, APC anti-mouse CD80, and PE-Cy7 anti-mouse CD86; mature DCs were recognized by CD11c^+^CD80^+^CD86^+^. DCs in the tumor were stained with FITC anti-mouse CD11c, PerCP anti-mouse CD45, PE-Cy7 anti-mouse CD80; mature DCs were recognized by CD45^+^CD11c^+^CD80^+^.

***Immunodeficient mouse model***

50 μL of PBS containing 2×10^6^ CT26 cells was subcutaneously injected into the right leg of the male Rag1-KO mice. The CT26 tumor-bearing mice were divided randomly into two groups: (1) control; (2) Se-*Ssp* (*i. t.*, 2×10^8^ /mL, 50 μL). The tumor length and width were recorded every two days to calculate the tumor volume by the formula Volume = Length × Width^2^/2. The tumors were collected on day 10, followed by homogenization into single-cell suspensions according to the recommended protocol. Then, these cell suspensions were stained with different antibodies and subjected to flow cytometry analysis. All cell samples were first blocked with anti-mouse CD16/32 antibody. DCs in the tumor were stained with FITC anti-mouse CD11c, PerCP anti-mouse CD45, PE-Cy7 anti-mouse CD80; mature DCs were recognized by CD45^+^CD11c^+^CD80^+^.

50 μL of PBS containing 2×10^6^ CT26 cells was subcutaneously injected into the right leg of the NOD/scid IL2rgnull mice. The CT26 tumor-bearing mice were divided randomly into two groups: (1) control; (2) Se-*Ssp* (*i. t.*, 2×10^8^ /mL, 50 μL). The tumor length and width were recorded every two days to calculate the tumor volume by the formula Volume = Length × Width^2^/2.

***Combination with immune checkpoint inhibitors***

50 μL of PBS consisting of 2×10^6^ CT26 cells was subcutaneously injected into the right leg of the Balb/c mice, and 50 μL of PBS consisting of 0.4×10^6^ CT26 cells was subcutaneously injected into the left leg of the Balb/c mice. These bilateral CT26 tumor-bearing mice were randomly divided into four groups: (1) control; (2) Se-*Ssp* (*i. t.*, 2×10^8^ /mL, 50 μL); (3) αCTLA-4 (*i. v.*, 20 μg); and (4) Se-*Ssp* (*i. t.*, 2×10^8^ /mL, 50 μL) + αCTLA-4 (*i. v.*, 20 μg). The tumor length and width were recorded every two days to calculate the tumor volume by the formula Volume = Length × Width^2^/2. The tumors that were collected after 7 days of the last treatment and subjected to CD8 immunofluorescence staining.

***Combination with IDO inhibitors***

50 μL of PBS consisting of 2×10^6^ CT26 cells was subcutaneously injected into the right leg of the Balb/c mice, and 50 μL of PBS consisting of 0.4×10^6^ CT26 cells was subcutaneously injected into the left leg of the Balb/c mice. These bilateral CT26 tumor-bearing mice were randomly divided into four groups: (1) control; (2) Se-*Ssp* (*i. t.*, 2×10^8^ /mL, 50 μL); (3) NLG919 (*i. g.*, 10 mg/kg); and (4) Se-*Ssp* (*i. t.*, 2×10^8^ /mL, 50 μL) + NLG919 (*i. g.*, 10 mg/kg). The tumor length and width were recorded every two days to calculate the tumor volume by the formula Volume = Length × Width^2^/2.

***Perfusion of bladder cancer***

25 μL of PBS consisting of 2×10^5^ MB49-luc cells was injected into the bladder wall of the C57BL/6 mice. These MB49-luc bladder tumor-bearing mice were randomly divided into three groups: (1) control; (2) BCG (*i. v. c.*, 50 mg/mL, 100 μL); (3) Se-*Ssp* (*i. v. c.*, 2×10^8^ /mL, 100 μL) and imaged by IVIS® Lumina III once every five days from day 5, with D-luciferin potassium salt as the substrate.

25 μL of PBS consisting of 2×10^5^ MB49 cells was injected into the bladder wall of the C57BL/6 mice. These MB49 bladder tumor-bearing mice were randomly divided into three groups: (1) control; (2) BCG (*i. v. c.*, 50 mg/mL, 100 μL); (3) Se-*Ssp* (*i. v. c.*, 2×10^8^ /mL, 100 μL). These bladders were collected on day 7, followed by homogenization into single-cell suspensions according to the recommended protocol. Then, these cell suspensions were stained with different antibodies and subjected to flow cytometry analysis. All cell samples were first blocked with anti-mouse CD16/32 antibody. DCs in the tumor were stained with FITC anti-mouse CD11c, APC anti-mouse CD80, PerCP anti-mouse CD45, and PE-Cy7 anti-mouse CD86; mature DCs were recognized by CD45^+^CD11c^+^CD80^+^CD86^+^. T cells in the tumor were stained with FITC anti-mouse CD3, PE anti-mouse CD8a, PerCP anti-mouse CD45, and PE-Cy7 anti-mouse CD4; CTLs were recognized by CD45^+^CD3^+^CD8a^+^, and Th cells were recognized by CD45^+^CD3^+^CD4^+^. Tregs in the tumor were stained with FITC anti-mouse CD3, PE anti-mouse FOXP3, PerCP anti-mouse CD45, and PE-Cy7 anti-mouse CD4; Tregs were recognized by CD45^+^CD3^+^CD4^+^FOXP3^+^. The supernatants from the bladder tissues were collected to measure the relative levels of cytokines via ELISA kits, including TNF-α (Invitrogen, USA) and IL-6 (Invitrogen, USA). The bladders were subjected to H&E and immunofluorescence staining.

***Statistical analysis***

All repeated experiments were performed using independent biological replicates. Data are presented as the mean ± SD. Statistical analysis was conducted using GraphPad Prism software. Statistical differences in survival were measured by the log-rank test. Statistical significance was calculated via unpaired two-tailed Student's t-test and expressed as ns *p* > 0.05, **p* < 0.05, ***p* < 0.01, and, ****p* < 0.001.

2. **Source**

**Table S1.** The information of antibodies

| **Antibody** | **Manufacturer** | **Clone** | **Cat. Number** |
| --- | --- | --- | --- |
| **Fluorochrome-conjugated Ab** | | | |
| Anti-mouse CD16/32 | Elabscience, China | 2.4G2 | E-AB-F0997A |
| FITC anti-mouse CD3e | Invitrogen, USA | 145-2C11 | 11-0031-85 |
| FITC anti-mouse-CD11c | Biolegend, USA | N418 | 117306 |
| FITC anti-mouse CD44 | Invitrogen, USA | IM7 | 17-0441-82 |
| FITC anti-mouse F4/80 | Biolegend, USA | BM8 | 123108 |
| FITC anti-mouse Gzmb | Biolegend, USA | GB11 | 515403 |
| PE anti-mouse CD3e | BD Bioscience, USA | 145-2C11 | 553030 |
| PE anti-mouse CD8a | Invitrogen, USA | 53-6.7 | 12-0081-82 |
| PE anti-mouse CD11b | Biolegend, USA | M1/70 | 101208 |
| PE anti-mouse CD40 | Biolegend, USA | 3/23 | 124610 |
| PE anti-mouse CD80 | Invitrogen, USA | 16-10A1 | 12-0801-82 |
| PE anti-mouse CD206 | Elabscience, China | C068C2 | E-AB-F1135D |
| PE anti-mouse MHC II | Invitrogen, USA | M5/114.15.2 | 12-5321-82 |
| PE anti-mouse CD274 | Biolegend, USA | 10F.9G2 | 124308 |
| PE anti-mouse FOXP3 | Biolegend, USA | MF-14 | 126404 |
| APC anti-mouse CD62L | Elabscience, China | MEL-14 | E-AB-F1011E |
| APC anti-mouse CD80 | Invitrogen, USA | 17-0801-82 | 16-10A1 |
| PerCP anti-mouse CD3 | Elabscience, China | 17A2 | E-AB-F1013F |
| PerCP anti-mouse CD8a | Biolegend, USA | 53-6.7 | 100732 |
| PerCP anti-mouse CD45 | Biolegend, USA | 30-F11 | 103130 |
| PE-Cy7 anti-mouse CD4 | Elabscience, China | GK1.5 | E-AB-F1097UH |
| PE-Cy7 anti-mouse CD45 | Elabscience, China | 30-F11 | E-AB-F1136UH |
| PE-Cy7 anti-mouse CD80 | Elabscience, China | 16-10A1 | E-AB-F0992UH |
| PE-Cy7 anti-mouse CD86 | Biolegend, USA | GL-1 | 105014 |
| PE-Cy7 anti-mouse CD206 | Elabscience, China | C068C2 | E-AB-F09832H |
| **Primary Ab** | | | |
| STING Rabbit mAb | Cell Signal Technology, USA | D2P2F | 13647S |
| Phospho-STING (Ser 365) Rabbit mAb | Cell Signal Technology, USA | D8F4W | 72971 |
| TBK-1/NAK | Cell Signal Technology, USA | E8I3G | 38066S |
| Phospho-TBK-1/NAK (Ser172) Rabbit mAb | Cell Signal Technology, USA | D52C2 | 5483S |
| IRF-3 Rabbit mAb | Cell Signal Technology, USA | D83B9 | 4302S |
| Phospho-IRF-3 (Ser396) Rabbit mAb | Cell Signal Technology, USA | D6O1M | 29047S |
| SELK Rabbit pAb | Proteintech, China | / | 12561-1-AP |
| IκBα Rabbit pAb | Affinity Bioscience, China | / | AF5002 |
| Phospho-IκBα (Ser32/Ser36) Rabbit pAb | Affinity Bioscience, China | / | AF2002 |
| NF-κB p65 Rabbit pAb | Affinity Bioscience, China | / | AF5006 |
| Phospho-NF-κB p65 (Ser536) Rabbit pAb | Affinity Bioscience, China | / | AF2006 |
| α-Tubulin Mouse mAb | Proteintech, China | 1E4C11 | 66031-1-Ig |
| β-Actin Rabbit rAb | Proteintech, China | 4H1 | 81115-1-RR |
| GAPDH Mouse mAb | Proteintech, China | 1E6D9 | 60004-1-Ig |
| **Secondary Ab** | | | |
| HRP-conjugated Goat Anti-mouse IgG rAb | Proteintech, China | / | RGAM001 |
| HRP-conjugated Goat Anti-rabbit IgG rAb | Proteintech, China | / | RGAR001 |
| AF647-labeled Goat Anti-rabbit IgG rAb | Beyotime Biotechnology, China | / | P0180 |
| **Therapeutic Ab** | | | |
| Anti-mouse CTLA-4 | BioXCell, USA | 9D9 | BE0164 |

3. **Supporting figures**

**Figure S1. The synthesis and characterization of Se-*Ssp*. (a)** Larger-scale culture of Se-*Ssp*. **(b)** The protein analysis around 50~70 kDa. **(c)** The hydrodynamic sizes of *Ssp* and Se-*Ssp* (n=3). **d-i. (d)** XPS survey spectra, **(e)** EDX spectra, **(f)** elemental mapping of N, O, C, and S, **(g)** Zeta potential (n=3), **(h)** the hydrodynamic size (n=3), and **(i)** protein concentration of the purified Se NPs (n=3). **(j)** The optical absorption of *Ssp* cultured in BG11 containing Na_2_SeO_3_ on days 0, 2, 4, 6, and 8 in the dark. **k-l.** Relative cell viability of **(k)** normal cell lines RAW264.7 (n=4), HUVEC (n=4), and **(l)** cancer cell lines CT26 (n=3), RM-1 (n=3), 4T1 (n=3) and MB-49 (n=3) incubated with Se-*Ssp* for 12 h. **(m)** The mean fluorescence intensity (MFI) of FITC (n=10). Data are presented as the mean ± SD. Statistical significance was calculated via unpaired two-tailed Student's t-test and expressed as ns *p* > 0.05, **p* < 0.05, ***p* < 0.01, and ****p* < 0.001.

**Figure S2. The** **immunostimulatory effects of Se-*Ssp*. (a)** Representative flow dot plots of MHC II expression on DCs stimulated with LPS, *Ssp,* and Se-*Ssp*. **(b)** The quantification of CD40 expression of DCs stimulated with LPS, *Ssp,* and Se-*Ssp* (n=3). **c-d,** The quantification of **(c)** DC maturation (n=3) and **(d)** MHC II expression on DCs stimulated with LPS, debris of *Ssp,* and Se-*Ssp* (n=3)*.* **(e)** ELISA results of TNF-α, IL-6, and IL-1β in the culture media of DCs stimulated with LPS, debris of *Ssp,* and Se-*Ssp* (n=3). **f-h.** The quantification of **(f)** CD80^+^ M1-phenotype macrophages, **(g)** CD206^+^ M2-phenotype macrophages, and **(h)** the ratio of M1/M2 phenotype macrophages stimulated with LPS, *Ssp,* and Se-*Ssp* (n=3). Data are presented as the mean ± SD. Statistical significance was calculated via unpaired two-tailed Student's t-test and expressed as ns *p* > 0.05, **p* < 0.05, ***p* < 0.01, and ****p* < 0.001.

**Figure S3. The *in vitro* mechanism** **of** **immunostimulatory effects induced by Se-*Ssp*. (a)** Western blotting analysis of TBK1/NAK, Phospho-TBK1/NAK, IRF3, Phospho-IRF3. **(b)** The ROS levels incubated with *Ssp*, Se NPs, and Se-*Ssp* for 12 h (n=5). **(c)** The quantification of DC maturation stimulated with Na_2_SeO_3_, G-Se NPs, Se NPs, and Se-*Ssp* (n=3). **(d)** Zeta potential of G-Se NPs, Se NPs, and Se-*Ssp* (n=3). **(e)** Western blotting analysis of SELENOK. **(f)** The quantification of DC maturation stimulated with *Ssp*, Se NPs, and Se-*Ssp*, with or without pre-treatment with Nifedipine as a Ca channel antagonist (n=3). **(g)** The quantification of DC maturation stimulated with LPS, Mn^2+^, LPS+Mn^2+,^ and Se-*Ssp* (n=3). **(h)** Co-localization of NF-κB p65 with the cell nucleus. **(i)** The MFI of Alexa Fluor™ 647 (n=10). Data are presented as the mean ± SD. Statistical significance was calculated via unpaired two-tailed Student's t-test and expressed as ns *p* > 0.05, **p* < 0.05, ***p* < 0.01, and ****p* < 0.001.

**Figure S4. The *in vivo* immune evaluation** **in the CT26 subcutaneous tumor model. a-j,** Groups: control (n=5), *Ssp* (n=5), Se NPs (n=5), and Se-*Ssp* (n=5). **a-e,** the quantification of **(a)** ResDC maturation in TDLNs, **(b)** M2-phenotype macrophages, **(c)** T cells, **(d)** CTLs, **(e)** Th cells in tumors on day 3 after the last injection. **(f)** the quantification of M2-phenotype macrophages in tumors on day 7 after the last injection. **g-h,** Representative immunofluorescence images of **(g)** CD3 and **(h)** CD4 of tumor sections. **i-j,** the ELISA results for **(i)** IL-6 and **(j)** IL-10 in tumors on day 3 and day 7 after the last injection. Scale bar: 50 μm. Data are presented as the mean ± SD. Statistical significance was calculated via unpaired two-tailed Student's t-test and expressed as ns *p* > 0.05, **p* < 0.05, ***p* < 0.01, and ****p* < 0.001.

**Figure S5.** **The therapeutic outcomes of Se-*Ssp*** **in the CT26 subcutaneous tumor model. (a)** The hemolytic rate of Se-*Ssp*, using ddH_2_O as a positive control (n=3). **b-c,** Groups: control (n=5), *Ssp* (n=5), Se NPs (n=5), and Se-*Ssp* (n=10). **(b)** Representative photographs of CT26 tumor-bearing mice treated with *Ssp*, Se NPs, and Se-*Ssp*. **(c)** The average body weight. **c-e,** Groups: control (n=5) and Se-*Ssp* (n=5). **d-f,** the quantification of **(d)** Tem and **(e)** Tcm in Th cells. **(f)** Representative flow dot plots of Tem and Tcm in Th cells. Data are presented as the mean ± SD. Statistical significance was calculated via unpaired two-tailed Student's t-test and expressed as ns *p* > 0.05, **p* < 0.05, ***p* < 0.01, and ****p* < 0.001.

**Figure S6. The *in vivo* mechanism of immunostimulatory effects induced by Se-*Ssp*** **in the CT26 subcutaneous tumor model. a-e,** Groups: control (n=5), Se-*Ssp* (n=5), Se-*Ssp*+Resatorvid (n=5), and Se-*Ssp*+CCCP (n=5). **(a)** The tumor volumes of individual mice. **(b)** The average body weight. **c-d,** the quantification of **(c)** DC maturation and **(d)** T cells in tumors. **(e)** Photographs of tumors. Data are presented as the mean ± SD. Statistical significance was calculated via unpaired two-tailed Student's t-test and expressed as ns *p* > 0.05, **p* < 0.05, ***p* < 0.01, and ****p* < 0.001.

**Figure S7.** **The therapeutic outcomes of Se-*Ssp* in the CT26 subcutaneous tumor model on immunodeficient mice. a-e,** Rag-KO mice, groups: control and Se-*Ssp*. **(a)** The tumor volumes of individual mice. **(b)** The average body weight (n=3). **(c)** The average tumor weight (n=4). **(d)** The quantification of DC maturation in TDLNs (n=5). **(e)** Representative immunohistochemical images of TUNEL of tumor sections. **f-h,** NSG mice, groups: control and Se-*Ssp*. **(f)** The tumor volumes of individual mice. **(g)** The average body weight (n=5). **(h)** The average tumor weight (n=5). **i-j,** Photographs of tumors from **(i)** Rag-KO mice (n=4) and **(j)** NSG mice (n=5). Scale bar: 100 μm. Data are presented as the mean ± SD. Statistical significance was calculated via unpaired two-tailed Student's t-test and expressed as ns *p* > 0.05, **p* < 0.05, ***p* < 0.01, and ****p* < 0.001.

**Figure S8. The combination of Se-*Ssp* with αCTLA-4 or NLG919 in the CT26 subcutaneous tumor model. a-b,** Groups: control (n=5), Se-*Ssp* (n=5), αCTLA-4 (n=5), and Se-*Ssp*+αCTLA-4 (n=10). The **(a)** primary and **(b)** distant volumes of individual mice. **c-e,** Groups: control (n=5), Se-*Ssp* (n=5), NLG919 (n=5), and Se-*Ssp*+NLG919 (n=5). **c-d,** The **(c)** primary and **(d)** distant volumes of individual mice. **(e)** The average body weight. **(f)** The overall survival rates. Data are presented as the mean ± SD. Statistical significance was calculated via unpaired two-tailed Student's t-test and expressed as ns *p* > 0.05, **p* < 0.05, ***p* < 0.01, and ****p* < 0.001.

**Figure S9. The therapeutic outcomes of Se-*Ssp* in the MB-49 orthotopic bladder tumor model. a-f,** Group: Control, BCG, Se-*Ssp*. **(a)** Photographs of the bladder with MB-49 orthotopic tumors. **(b)** Representative images of B-mode ultrasound scans. **c-d,** The quantification of **(c)** Th cells (n=5) and **(d)** Tregs in MB-49 orthotopic bladder tumors (n=5). **(e)** Representative H&E staining of MB-49 orthotopic bladder tumor sections. **(f)** Representative immunofluorescence images of CD8 in MB-49 orthotopic bladder tumor sections. **(g)** H&E staining of urothelium, lamina propria, and muscularis propria of bladder after perfusion with Se-*Ssp*. Scale bar: 50 μm. Data are presented as the mean ± SD. Statistical significance was calculated via unpaired two-tailed Student's t-test and expressed as ns *p* > 0.05, **p* < 0.05, ***p* < 0.01, and ****p* < 0.001.
